# The effect of water on colloidal quantum dot solar cells

**DOI:** 10.1038/s41467-021-24614-7

**Published:** 2021-07-19

**Authors:** Guozheng Shi, Haibin Wang, Yaohong Zhang, Chen Cheng, Tianshu Zhai, Botong Chen, Xinyi Liu, Ryota Jono, Xinnan Mao, Yang Liu, Xuliang Zhang, Xufeng Ling, Yannan Zhang, Xing Meng, Yifan Chen, Steffen Duhm, Liang Zhang, Tao Li, Lu Wang, Shiyun Xiong, Takashi Sagawa, Takaya Kubo, Hiroshi Segawa, Qing Shen, Zeke Liu, Wanli Ma

**Affiliations:** 1 Institute of Functional Nano and Soft Materials (FUNSOM), Jiangsu Key Laboratory for Carbon-Based Functional Materials and Devices, Joint International Research Laboratory of Carbon-Based Functional Materials and Devices, Soochow University, Suzhou, Jiangsu China; 2 Research Center for Advanced Science and Technology, The University of Tokyo, Meguro-ku, Tokyo Japan; 3 Faculty of Informatics and Engineering, The University of Electro-Communications, Tokyo, Japan; 4 Department of Chemistry and Biochemistry, Northern Illinois University, DeKalb, IL USA; 5 X-ray Science Division, Argonne National Laboratory, Lemont, IL USA; 6 Graduate School of Energy Science, Kyoto University, Kyoto, Japan

**Keywords:** Electronic devices, Quantum dots

## Abstract

Almost all surfaces sensitive to the ambient environment are covered by water, whereas the impacts of water on surface-dominated colloidal quantum dot (CQD) semiconductor electronics have rarely been explored. Here, strongly hydrogen-bonded water on hydroxylated lead sulfide (PbS) CQD is identified. The water could pilot the thermally induced evolution of surface chemical environment, which significantly influences the nanostructures, carrier dynamics, and trap behaviors in CQD solar cells. The aggravation of surface hydroxylation and water adsorption triggers epitaxial CQD fusion during device fabrication under humid ambient, giving rise to the inter-band traps and deficiency in solar cells. To address this problem, meniscus-guided-coating technique is introduced to achieve dense-packed CQD solids and extrude ambient water, improving device performance and thermal stability. Our works not only elucidate the water involved PbS CQD surface chemistry, but may also achieve a comprehensive understanding of the impact of ambient water on CQD based electronics.

## Abstract (web summary)

Surface of colloidal quantum dot is sensitive to water, and the interaction could potentially alter its chemical environments. Here, Shi et al. investigate how the interaction effects the nanostructures and carrier dynamic in CQDs, and subsequently introduce meniscus-guided coating technique to mitigate CQD fusion triggered by water adsorption.

## Introduction

The presence of water on solid surfaces is ubiquitous in nature, which significantly impacts the surface chemical process and the corresponding properties of metals, oxides, and semiconductors through surface hydroxylation and water adsorption^1–3^. The surface-dominant nature of colloidal quantum dots (CQDs) endows them extreme surface sensitivity towards ambient humidity^4^. It has been reported that humid ambient has significant impacts on the properties of CQDs^5–8^. Uncovering the effect of water should be essential to open the door towards the ambient manufacturing of CQD-based electronic devices. However, the water-involved surface chemistry and its potential influence on CQD electronics have rarely been explored yet.

Semiconducting lead sulfide (PbS) CQDs are promising building blocks for solution-processed electronics, including photovoltaics, infrared photodetectors, and field-effect transistors^9,10^. The surface geometry plays a critical role in the CQD surface chemistry and performance of devices, which can be predicted by Wulff constructions based on surface energy minimization theorem^11,12^. The surface of PbS CQD with a small size of less than 3 nm is dominated by polar {111} facet. Further growth of CQDs will lead to the appearance of {100} and {110} facets. This feature of CQD with controllable surface geometries provides a model platform for the study of the water effect on CQD characteristics, starting from the atom-level surface chemistry to the macroscopic photovoltaic properties.

Generally, the environmental water molecules can hydroxylate the solid surface via dissociative chemisorption during material synthesis or ambient device fabrication^13,14^. In 2014, Zherebetskyy et al. demonstrated that the dissociated water molecules could cause surface hydroxylation during PbS CQDs synthetic process^15^. The interposition of the hydroxyl anions (OH) between steric oleic acid (OA) ligands effectively releases the surface energy of {111} facet, preserving facet stabilization. The strong bonding between surface Pb and the hydroxyls on {111} facet make OH hard to be removed by the conventional ligand exchange process^16^. The discovery of partially hydroxylated surfaces has deepened the understanding of the surface science in PbS CQDs. Thereafter, close associations between the surface OH and the deficiency in solar cell performance have been observed. Cao et al. achieved improvement in power conversion efficiency (PCE) and photostability of solar cells based on iodine-passivated PbS (PbS-I) CQD by effectively reducing the surface OH ligands^17^. Followed by this innovation, strategies were developed to avoid the adverse effect of surface hydroxyls, including precursor engineering^16^, direct synthesis of CQD inks^18^, protic solvent control^19^, ink engineering^20^, and the introduction of etching agent^21–23^. Although the investigation results suggest that the surface OH may lead to trap states, the identification of involved hydroxyl species and their exact connections with the defectiveness in PbS CQDs are still substantial subjects of debate^11,24,25^. So far, there is no theoretical or experimental evidence to support that the surface OH ligands could directly serve as the trap sources. Thus, it remains an urgent challenge to understand the pathways from ambient water to PbS surface hydroxylates and eventually to the reduced device performance.

Subsequently, the hydroxylation on solid surfaces allows the adsorption of ambient water by hydrogen bonding between the surface OH and the water molecules, forming a configuration of mixed OH + (H_2_O)_*n*_^26–28^. The adsorbed water on solid surfaces with a thickness ranging from a few Å to infinite bulk may participate in surface chemical reactions^29^. However, the water adsorption on hydroxylated PbS CQDs under humid air has been ignored so far. Thus, the investigation of adsorbed water may shed new light on the surface chemistry and the mystery of hydroxyl-induced device deficiency in PbS CQD solar cells.

In this study, H-bonded water is identified for the first time on the partially hydroxylated surface of PbS CQDs by in-situ temperature-dependent X-ray photoelectron spectroscopy (XPS), X-ray absorption spectroscopy (XAS), and the density functional theory (DFT) studies. The results indicate that the adsorbed water could govern the temperature-dependent change of CQD surface chemistry through water desorption, hydroxylation, and dehydration process. More importantly, we reveal that exposure to ambient water during device processing will enhance the surface hydroxylation and water adsorption, which subsequently aid the fusion of adjacent CQDs. The inter-band trap states and energetic disorder introduced by fused CQDs will harm the performance of CQD solar cells, which unambiguously elucidate the correlation between surface OH ligands and device deficiency. We also reveal that the notorious thermal instability of PbS CQD solar cells may originate from the continuous fusion, as well as surface iodine loss and migration during thermal aging. To resolve the issue, the meniscus-guided deposition technique, i.e., convective assembly (CA), was used instead of spin-coating to achieve morphological homogeneity of CQD films. Resultantly, the densely packed CQD arrays could spatially extrude the ambient water and thus reduce surface hydroxylates, leading to improved device performance and thermal stability. We believe our works not only elucidate hydroxyl involved CQD surface chemistry but also achieve a comprehensive understanding of the impact of ambient water on CQD solar cells, which may provide general insight into the practical manufacturing of stable CQD based electronics via a scalable manner under ambient air.

## Results

### Identification of surface hydroxylates

**Fig. 1:**
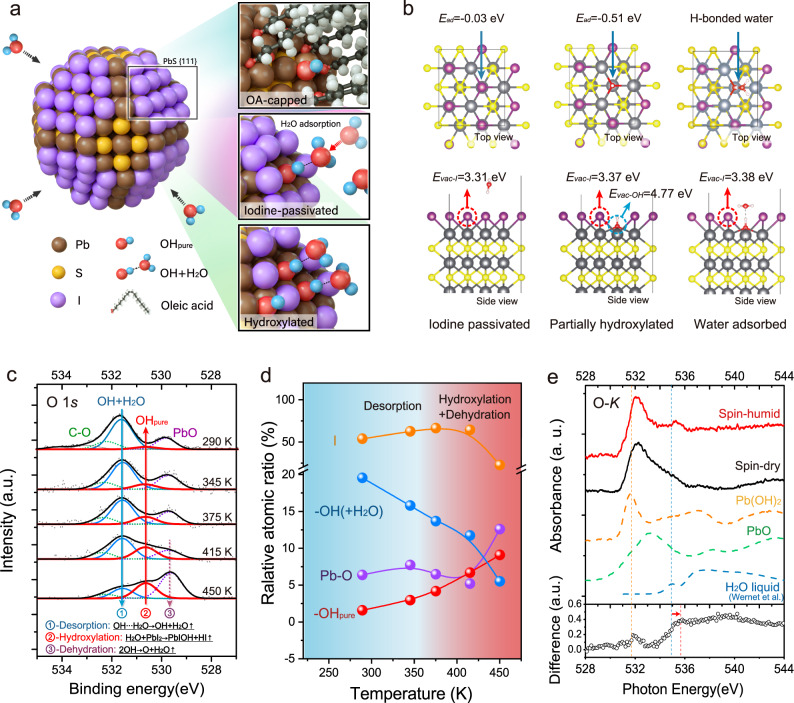
Identification of water adsorption on partially hydroxylated CQD surfaces. **a** Schematic representation of surface conditions on octahedral PbS CQDs. The ideal model of PbS CQDs with atomic halide passivation is shown on the left. The proposed surface conditions on the Pb-terminated {111} facet are zoomed on the right. Top: the OA-capped facet with partial surface hydroxylation introduced in the synthetic process^15^; Medium: Atomic iodine-passivated facet after ligand exchange; Below: the aggravation of surface hydroxylation followed by water adsorption under humid air. **b** The geometric structures of iodine-passivated (left), partially hydroxylated (middle) and water adsorbed PbS {111} facets (right) used in DFT calculation. The *E*_ad_ and *E*_vac_ stand for the adsorption energy of H_2_O and the vacancy formation energy, respectively. The purple spheres stand for iodine atoms, black ones for Pb atoms, yellow ones for S atoms, red ones for O atoms, and white ones for H atoms. **c** The temperature-dependent O1*s* XPS spectra of PbS-I film. **d** Atomic ratio of surface species relative to Pb 4*f* core recorded at a temperature from 290 K to 450 K. **e** XAS spectra of PbS CQD films at the oxygen K-edge prepared under different ambient conditions. The “Spin-humid” and “Spin-dry” represent the sample deposited and ligand exchanged under ambient air with a high RH (50–60%) or low RH (<10%), respectively. The XAS spectra of liquid water and different surface chemical species observed in XPS O1*s* core are also shown. The XAS spectra of liquid water are extracted from Wernet et al. (2004)^2^. Reprinted with permission from AAAS. The spectra difference between “Spin-humid” and “Spin-dry” is calculated for a clearer comparison.

Figure 1a shows the geometry structure of truncated octahedron PbS CQD used in our study. The surfaces of ~3 nm PbS CQD used in our studies are mainly dominated by PbS {111} facets, with partial coverage of {100} facets. In ideal situations, the CQD should be fully covered by iodine on the polar {111} facet. However, the strong steric hindrance of OA allows partial surface hydroxylation, stabilizing PbS {111} facets during the synthesis process^15^. The surface OH could provide potential absorption sites for ambient water, which makes CQD more vulnerable to the ambient environment.

To theoretically predict the water adsorption on CQD surfaces, we employ the slab models including PbS {111}, {100}, and {110} facets covered by different surface species as the geometric configurations in our density functional theory (DFT) calculations, as illustrated in Fig. 1b and Supplementary Fig. 1^11^. By calculating the adsorption energy (*E*_ad_) between water molecules and CQD surfaces, the most stable adsorbate configuration can be revealed^30^. The *E*_ad_, density of states (DOS), and bonding motifs of {111} surfaces are displayed and summarized in Supplementary Fig. 2 and Supplementary Table 1. For the fully iodine-passivated PbS facet, a negligible *E*_ad_ suggests no capability for water absorption. As for the partially hydroxylated facets, preferable water adsorption on surface OH ligands can be predicted by a low *E*_ad_ of −0.51 eV per H_2_O. A clean band edge without mid-gap states introduced by surface OH or H-bonding is suggested by DOS in Supplementary Fig. 3, which indicates that OH or adsorbed water molecules will not directly introduce surface trap states. It is worth noting that the oxidized facet exhibits the strongest capability of water adsorption (Supplementary Fig. 2c). The large electronegativity of oxygen atom may pull the hydrogen in water molecule to the neighbor Pb-O site, forming two hydroxyls through a reverse OH recombination reaction^31^.

To experimentally identify the surface hydroxylates, systematical X-ray spectroscopy studies were performed. The in-situ temperature-dependent XPS measurement provides experimentally traceable clues for the water chemistry on the CQD surface, where the heating under ultra-high vacuum (UHV) could kinetically overcome the activation barrier for a specific chemical reaction^32^. Figure 1c illustrates the temperature-dependent O 1 *s* spectra of iodine-passivated PbS CQD film prepared by solid-state-ligand-exchanged (SSLE) method measured at 290–450 K. The sample was spin-coated under ambient condition (40–50% relative humidity). The center peak with the binding energy (BE) at 531.6 eV (acquired at 290 K) is generally assigned to oxygen in OH-groups, the peak centered at 529.8 eV to Pb–O, and the high BE shoulder to C–O species^33^. However, with increasing temperature, a new peak at 530.7 eV emerges and the OH peak at 531.6 eV decreases. The unexpected peak evolution suggests that the OH-related surface species on PbS CQDs are more complex than originally assumed in literatures, as summarized in Supplementary Table 3^15^. Similar spectra change have been observed in the in-situ temperature-dependent XPS studies of water adsorption on metal surfaces^32^. We thus reassign the original OH peak at 531.6 eV to the mixed OH + H_2_O phases formed by hydrogen bonding between the surface OH and water molecules, as supported by our DFT results. Consequently, we ascribe the new peak emerging at 530.7 eV with increasing temperature to the pure hydroxyl groups on PbS CQD surfaces due to water desorption, noted as OH_pure_.

For the partially hydroxylated PbS CQD surface with iodide passivation, we reveal that water plays a critical role in the thermally induced evolution of surface chemistry under UHV by three steps: (1) The desorption of surface absorbed water; (2) the aggravation of surface hydroxylation; (3) the dehydration of surface hydroxyls. Figure 1d shows the atomic ratio of surface species relative to Pb 4*f* with increasing temperatures from 290 K to 450 K. The corresponding XPS spectra and atom ratio are summarized in Supplementary Fig. 4 and Supplementary Table 4. Briefly, a trade-off between OH_pure_ and OH + H_2_O species on the CQD surface is observed, which could proceed through a desorption process of H-bonded water by:

1 $${\rm{OH}}\cdots {{\rm{H}}}_{2}{\rm{O}}\mathop{\longrightarrow }\limits^{\varDelta }{{\rm{OH}}}_{{\rm{pure}}}+{{\rm{H}}}_{2}{\rm{O}}\uparrow ({\rm{Desorption}})$$

Upon transition of surface hydroxylates from mixed OH + H_2_O phases to OH_pure_ at high temperature_,_ the surface iodide decomposes quickly, as reflected by the sharply reduced I/Pb ratio at high temperature (Supplementary Table 4)^34–36^. The serious iodine loss may partially stem from the thermally induced Schottky vacancies. It is also likely that the remaining water will participate in a basic salt reaction at a high temperature above 415 K:

2 $${{\rm{H}}}_{2}{\rm{O}}+{{\rm{PbI}}}_{2}\mathop{\longrightarrow }\limits^{\varDelta }{\rm{PbIOH}}+{\rm{HI}}\uparrow ({\rm{Hydroxylation}})$$

In this case, the adsorbed water on the CQD surface may not merely work as a “spectator”, namely, being extracted into the vacuum after desorption, but rather work as the reactants in hydroxylation process, significantly replacing the iodine by OH_pure_. Similar chemical evolution has been identified in the water-associated decomposition process of lead halide perovskite, forming a subsalt of lead hydroxide iodide (PbIOH) under humid ambient^37^. Meanwhile, the OH_pure_ ligands could form the Pb–O species via an OH recombination reaction at elevated temperatures, with the resultant atomic O coordinated with neighbor Pb on facets:^38^

3 $${{\rm{OH}}}_{{\rm{pure}}}+{{\rm{OH}}}_{{\rm{pure}}}\mathop{\longrightarrow }\limits^{\varDelta }{\rm{O}}+{{\rm{H}}}_{2}{\rm{O}}\uparrow ({\rm{Dehydration}})$$

The Pb–O accumulation and I loss are consistent with the results in Fig. 1d. The in-situ temperature-dependent XPS unveils the CQD surface chemistry under UHV conditions, which indicates the critical role of adsorbed water in the evolution of surface species.

We found that the surface hydroxylation not only happened during the synthesis process but could also occur during device fabrication under humid air. The ex-situ XPS measurements of CQD films with or without annealing under dry or humid ambient are conducted, as shown in Supplementary Fig. 5 and Supplementary Table 5. With the aid of gentile annealing, drastic growth of surface hydroxylates under the humid atmosphere is obtained. A spontaneous reaction can be involved:

4 $$2{{\rm{H}}}_{2}{\rm{O}}+{{\rm{PbI}}}_{2}+\frac{1}{2}{{\rm{O}}}_{2}\to 2{\rm{Pb}}{({\rm{OH}})}_{2}+{{\rm{I}}}_{2}\uparrow ({\rm{Hydroxylation}}\,{\rm{under}}\,{\rm{humid}}\,{\rm{air}})$$

This reaction on CQD PbS {111} facet is theoretically supported by the reduction of total potential energy from DFT calculations (Δ*E* = −94.40 kJ/mol), as shown in Supplementary Fig. 6a, suggesting reaction (4) is energetically preferable on CQD surfaces. The resultant surface hydroxyls can be stabilized by the following H-bonding formation with adsorbed water, further reducing the system energy by −48.96 kJ/mol. The theoretical prediction agrees well with the XPS experimental results, as shown in Supplementary Fig. 6c-d. The continuous heating of PbS-I CQD film leads to nearly full-surface hydroxylation and I loss. A similar chemical routine is also suggested as one of the decay pathways of lead halide perovskites under humid ambient^39^.

To confirm the water adsorption and further understand the water bonding motifs on hydroxylated PbS CQD surface, O K-edge X-ray spectra (XAS) are measured and shown in Fig. 1e. The XAS has been extensively used to detect the water configuration due to its high sensitivity to the H-bond distortions on the H-sides of water molecules^2,40,41^. The O K-edge XAS spectra of Pb(OH)_2_, PbO, and liquid H_2_O are presented as references^2^. A dominant pre-edge feature is observed between 531 eV to 532 eV in the XAS spectrum of the spin-coated PbS-I samples prepared under dry air, which is mainly contributed by Pb–O and Pb–OH species. For the sample prepared under humid air (RH ~50–60%), a new peak around 535.3 eV emerges, attributed to the adsorbed surface water. The distinction of surface species between spin-dry and spin-humid samples can be further identified in the difference spectrum (lower row of Fig. 1e). The sharp peak at 531.9 eV can be ascribed to the difference in Pb–OH surface species varied by ambient humidity. The notable peak near 535.5 eV is caused by water adsorption, while it shows an energy shift of 0.7 eV relative to the peak near the pre-edge of liquid water (blue dashed line, Fig. 1e). Generally, the liquid water molecules exhibit local coordination with only one strongly H-bonded OH group, reflected by a distinct pre-edge feature in XAS, whereas ice shows fully coordinated hydroxyls characterized by XAS spectra with only post-edge peak^2^. Thus, the peak shift in the difference spectra indicates that a certain amount of adsorbed water molecules may form two hydrogen bonding with the surrounding OH, transforming from a single donor (liquid) to double donor (ice) configuration on the CQD surface^2,40^.

Resultantly, a schematic surface illustration of truncated octahedral PbS CQD is shown in Fig. 1a. The hydroxylates on PbS CQD surfaces can be formed under ambient conditions via three routes: (1) the strong steric hindrance of OA allows partial surface hydroxylation to stabilize the PbS {111} facet during the synthesis process (Supplementary Fig. 7, formula S-1)^15^. The hydroxyls are hard to be removed during the ligand exchange process due to the strong Pb–OH bonding^16^; (2) the exposure of hydroxylated surfaces to the ambient water, leading to water adsorption by H-bonding (formula S-2, S-7); (3) The hydroxylation on iodine-passivated CQD facets during ambient processing accelerated by gentile annealing, along with the subsequent surface adsorption of ambient water (formula S-6, S-7).

### Water impacted morphology in CQD solids

**Fig. 2:**
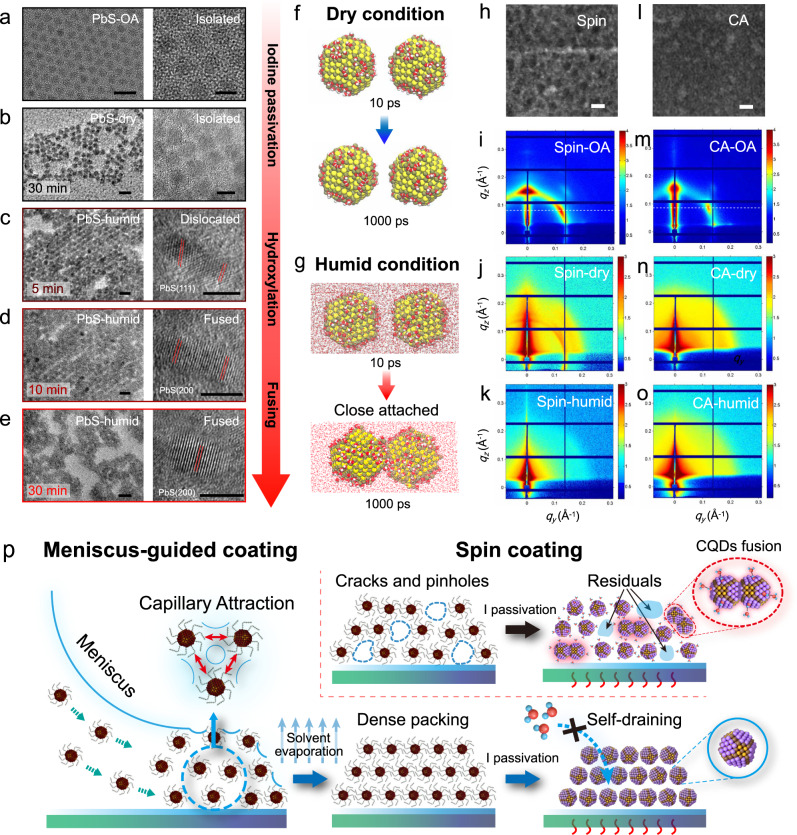
The impact of ambient water on the CQD nanostructure and film morphology. **a**–**e** The TEM images of PbS CQDs prepared under different conditions. The samples were prepared on carbon meshes by (**a**) droplet deposition (**b**) followed by ligand exchange and annealing process at 85 °C under dry air, (**c**–**e**) or under humid air with different annealing times. The scale bars: left column 10 nm and right column 5 nm. **f**, **g** Molecular dynamic simulation of the water effect on hydroxylated CQDs. Atom color: brown, Pb; yellow, S; red, O; white, H. Snapshots of two CQDs with surface hydroxyls covered on {111} facet with a gap distance of 1 nm at 300 K after (**f**) 10 ps and (**j**) 1000 ps. The ambient water could work as H-bonding bridges, which significantly promote the close attachment of neighboring CQDs. **h**, **l** The focused ion beam (FIB) cross-sectioned bright-field TEM images of CQD stacks prepared through (**h**) spin coating and (**l**) CA. Scale bars: 10 nm. The GISAXS patterns of CQD films prepared by (**i**) spin coating before ligand exchange and (**j**) after ligand exchange under dry air annealing or (**k**) under humid air annealing, or by (**m**) CA deposition before ligand exchange and (**n**) after ligand exchange under dry air annealing or (**o**) under humid air annealing. **p** The illustration of meniscus-guided coating and spin-coating process for the preparation of PbS CQD solids. The densely packed CA films show the self-draining effect against ambient water intrusion.

The ambient water can not only guide the surface chemistry process but also change the morphology in CQD solids at different spatial scales. Figure 2a–e demonstrates the high-resolution transmission electron microscope (TEM) images of PbS CQDs prepared under different conditions. Under dry air, the substitutions of long OA ligand by atomic iodine passivants shorten the inter-dot spacing but keep CQDs intact after heating for 30 min at 85 °C (Fig. 2b). In comparison, under humid air, apparent epitaxial CQD fusion along with increasing heating time is observed in Fig. 2c–e. In this case, hydroxylation and subsequent water adsorption on CQD surfaces can be aggravated, as presented in Supplementary Fig. 5d. Serious CQD fusion was also found in the heavily hydroxylated PbS CQDs treated by tetramethylammonium hydroxide pentahydrate (TMAOH·5H_2_O) methanol solution^42^ even without further annealing process (Supplementary Fig. 8c), suggesting the close link between surface hydroxylates and CQD fusion.

Generally, the CQDs should go through several steps before completely fusing into large crystals, including the CQD close attachment, rotations, planar alignments, and interfacial relaxation^43^. This process can be affected by multiple factors, including the van der Waals (vdW) attractions, entropic factors, and the atomic bonding of specific crystal facets^44^. In any case, the close attachment of neighboring CQDs is the prerequisite for fusion processing. We suppose that the water molecules displaced between hydroxylated CQD surfaces could work as H-bonding bridges, which provide extra inter-CQD attractions and thus facilitates CQD fusion. To testify our hypothesis, molecular dynamic (MD) simulations are performed and shown in Fig. 2f, g. Two individual hydroxylated CQDs are displaced with a gap distance of 1 nm at 300 K in the first place. After 1000 ps, almost no change in inter-CQD distance is found for the condition without ambient water. In sharp contrast, the CQDs under humid ambient rapidly attach with each other, which highlights the essential role of ambient water in CQD fusion. To understand the possible driving force for the close attachment assisted by water, we performed DFT calculations on face-to-face PbS {111} slabs with different passivation conditions, as shown in Supplementary Fig. 9. By replacing half of the surface iodine with hydroxyls bonded by ambient water, the relaxed inter-facet distance is remarkably reduced from 4.216 Å to 3.776 Å. The O in H_2_O could form two H-bonds with surface hydroxyls on neighboring {111} facets, linking the CQD together and promoting CQD close attachment. This result fits well with the peak shift observed in the XAS spectra (Fig. 1b), where the formation of the H-bonding bridges between neighboring CQDs may promote the transformation of adsorbed surface water from a single donor to double donor configuration, shifting the peak edge to higher energy. The closely attached CQDs have certain mobility under gentle annealing (far below the melting point of bulk crystals), promoting further rotations and fusion along {100} facet^43,45^.

In addition, we find that the morphology-governed water entrapping in PbS CQD solids can be affected by different deposition techniques. The traditional SSLE PbS-I CQD films spin-coated by layer-by-layer (LbL) method inevitably generate inhomogeneous film morphology with striation structures, as shown in the cross-sectional TEM images in Fig. 2h and Supplementary Fig. 10. The strong black-and-white contrast in the TEM image corresponds to the difference of electron scattering throughout the cross-sectional CQD slide with a thickness of ~100 nm, reflecting incompact bulk morphology inside CQD films^46,47^. The porous morphology in CQD solids may enhance water trapping and CQD surface hydroxylation. To reduce the impact of ambient water, a meniscus-guided-coating technique, i.e., convective assembly (CA) is introduced^48^. Benefiting from the strong capillary attraction among CQDs during the assembly process, a much more compact film can be achieved without apparent voids and organic striations (Fig. 2l), which reduces water preservation in CA films. To further understand the effect of water on film morphology at different spatial scales, the GISAXS patterns of CQD films fabricated under different conditions were collected and presented in Fig. 2i–k and Fig. 2m–o, with the relative azimuthally integrated patterns exhibited in Supplementary Fig. 11. The OA capped CQD films before ligand exchange shows a distinct diffraction ring, indicating an inter-CQD spacing of 5.18 nm (Fig. 2m) and 5.35 nm (Fig. 2i) for CA film and spin-coated film, respectively^49,50^. The shorter inter-dot spacing demonstrates the superior compactness of CA film. Ligand exchange reduces the packing order and broadens the diffraction pattern for both PbS-I films. The mingled rings in Fig. 2j indicate inter-spaces between 5.35 and 5.43 nm, likely due to the incompletely exchanged OA ligands or other organic residuals in spin-coated film. In comparison, uniform diffraction patterns can be observed in Fig. 2n confirming the improved homogeneity for close-packed CA film. More importantly, the morphology of CQD solids is found to be sensitive to ambient water. After annealing for 30 min under humid air, the GISAXs spectrum of spin-coated PbS-I films (Fig. 2k) changes significantly. The weak diffraction pattern suggests less ordered CQDs packing, where the inter-space is mainly governed by the large inter-domain distance between fused CQDs aggregations, as reflected by the TEM images in Fig. 2h, and illustrated in Fig. 2p (right). In sharp contrast, the pattern of CA film with stable surface morphology (Supplementary Fig. 12) shows no significant change towards different ambient RH (Fig. 2n–o), exhibiting strong water resistance. The inherent advantages of meniscus-guided assembly give rise to the self-draining effect to effectively extrude water residuals in CQD films, as illustrated in Fig. 2p (left), which minimizes the subsequent hydroxylation and water adsorption on the CQD surface as proved by the XPS (Supplementary Fig. 5e) and the XAS (Supplementary Fig. 13) results.

### Water impacted photoelectric properties of CQD solids

**Fig. 3:**
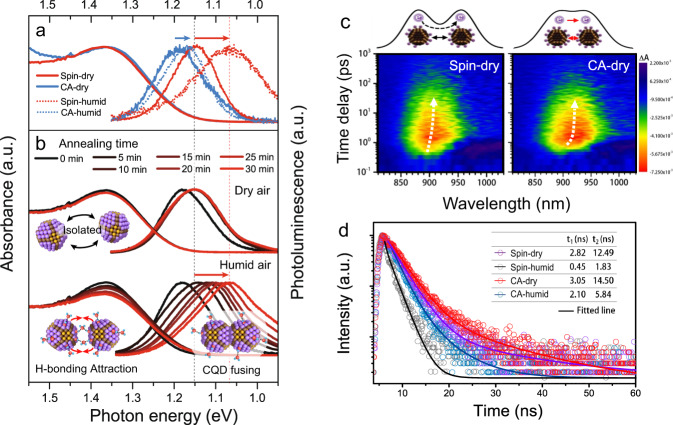
Spectroscopy and carrier dynamic studies of the CQD solids impacted by ambient water. **a** The absorbance, steady-state photoluminescence (PL) spectra of PbS-I CQD films prepared by spin coating and CA under different humidity. **b** The change of absorbance and PL spectra of spin-coated PbS-I CQD films versus heating time under dry and humid air. **c** Pseudo-color TA spectra of PbS-I CQD films by spin coating and CA. The color scales for a spin and CA films are the same and proportional to the absorption change in each graph. All samples were pumped by 470 nm laser pulse with the pump flux of 25 J·cm^−2^. **d** Transient PL decay of CQD films; inset table: the fast and slow time component fitted by double-exponential function.

The ambient water significantly affects the PbS CQD surface chemistry and morphology, consequently, changing the optical properties and carrier dynamics in CQD films. Figure 3a shows the absorption and photoluminescence (PL) spectra of PbS-I films prepared under different conditions followed by annealing at 85 °C for 30 min. The corresponding optical parameters are summarized in Table 1. A Stokes shift of 169 meV for the CA film prepared under dry air can be observed, while the spin-coated film shows a Stokes shift of 223 meV. The smaller Stokes shift can be ascribed to the better CQDs packing order in CA film, leading to flattened energetic landscape and improved charge carrier transport^51–53^. Resultantly, the CA film shows a rapid spectra diffusion in transient absorption spectra (TAS) at the time scale of several picoseconds (Fig. 3c, Supplementary Fig. 14), indicating the fast charge transfer due to enhanced electrical coupling in ordered CQD arrays (see Supplementary Note 1 for detailed explanations). In comparison, the inhomogeneous morphology of spin-coated film causes an uneven energetic landscape, impeding the charge transport and broadening the Stokes shift to a certain extent^54,55^. After exposure of the fabrication process to humid ambient (RH 50–60%), a drastically increased Stokes shift of 305 meV can be observed for the spin-coated film. In contrast, a Stokes shift of only 189 meV is observed for CA film, with a slight change of 20 meV induced by humidity variation. Note that in the previous studies, the surface vacancies in PbS CQD have been widely regarded as the source of trap states, which could vary the Stokes shift to more than hundreds of meV^36,56,57^. However, in our DFT results, the introduction of the surface hydroxylation or water adsorption does not reduce the vacancy formation energy of surface iodine (*E*_vac-I_ from 3.31 eV to 3.38 eV on {111} facet). Instead, the high vacancy formation energy of OH (*E*_vac-OH_ of 4.77 eV), as shown in Fig. 1b, makes the CQD surface vacancies thermodynamically hard to be formed^34^. (see Supplementary Note 2 for detailed explanations). Thus, a new mechanism may lie behind the discrepancy.

**Table 1:** Summarized photoelectric properties for the CQD solids prepared under different conditions.

Deposition Condition	First excitonic peak (meV)	PL peak position (meV)	Stokes shift (meV)	*CB* (eV)	*VB* (eV)	Electron mobility *m*^a^(cm^2^V^−1^s^−1^)	Trap state density *n*_trap_^a^ (cm^−3^)	PL lifetime^b^	
								*t*_*1*_ (ns)	*t*_*2*_ (ns)
Spin									
Dry air	1369	1146	223	−4.18	−5.57	9.38 × 10^−5^	1.76 × 10^16^	2.82	12.49
Humid air	1372	1067	305	−4.00	−5.42	5.47 × 10^−5^	3.21 × 10^16^	0.45	1.83
CA									
Dry air	1356	1187	169	−4.23	−5.62	1.77 × 10^−4^	1.72 × 10^16^	3.05	14.50
Humid air	1359	1170	189	−4.15	−5.54	8.51 × 10^−5^	2.10 × 10^16^	2.10	5.84

^a^The mobility and trap state density is calculated from the SCLC measurement in Supplementary Fig. 15.

^b^The lifetime is fitted from Fig. 3d by a second-order exponential model.

Our studies reveal that the Stokes shift is strongly correlated with the extent of CQD fusion and the corresponding hydroxylates amount on CQD surfaces. Figure 3b shows the absorbance and PL spectra of spin-coated PbS-I CQD films annealed under different ambient conditions. The films annealed under dry air exhibit a very limited peak shift in PL spectra, where the individual CQDs remained to be well-isolated (Fig. 2b). As a comparison, for the film heated under humid air, the PL peak red-shifts continuously with increasing annealing time, accompanied by the diminution of the first excitonic absorption peaks. This change in spectroscopy is well-fitted to the continuously intensified CQD fusion aided by ambient water as observed in Fig. 2c–e. The strong electrical coupling between fused CQDs generates new states lower in energy by 100–200 meV relative to the CQD bandgap^58–60^, creating inter-band traps that broaden the Stokes shift and reduce the PL lifetime (Fig. 3d)^61^. This result is well in accordance with the enhanced trap-filled limited voltage (*V*_TFL_) and trap states density (*n*_trap_) from the space-charge-limited current (SCLC) measurements in Supplementary Fig. 15. Thus, we owe the large Stokes shift in spin-coated CQD film under humid conditions to the serious CQD fusion, rather than the formation of surface Schottky vacancies. We found that the water-triggered fusion and related trap behavior in CQD devices are very general. The effect of water on atomic halide passivated CQD active layers prepared by three different methods are studied, including the solution-phase-ligand-exchanged (SPLE) inks and the direct-synthesized (DS) inks of PbS CQDs, as well as the SPLE PbSe CQD inks, as shown in Supplementary Fig. 16. Similar trends in surface conditions and optical properties can be concluded, exhibiting the generality of water-triggered fusion and related trap behavior in atomic halide passivated CQDs solids.

### Water affected solar cell performance and thermal stability

**Fig. 4:**
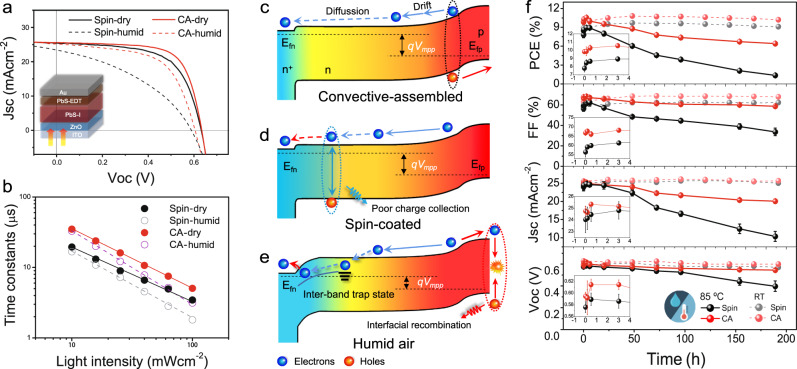
Solar cell performance of CQD devices intervened by ambient water. **a** *J−V* curves of CQD solar cells fabricated through the spin coating and CA under dry air and humid air (RH ~50%–60%), measured under AM 1.5 G solar simulator. **b** Light-intensity-dependent electron recombination lifetime constants for CQD devices. The relative Bode plots are shown in Supplementary Fig. 18. **c**–**e** Band diagrams for CQD cells at *V*_MPP_ condition for (**c**) CA devices and (**d**) spin-coated devices prepared under dry air and (**e**) under humid air, where *E*_*Fn*_ is the electron quasi-Fermi level, and *E*_*Fp*_ is the hole quasi-Fermi level. **f** Stability of CQD devices under room temperature or under continuously heating at 85 °C in ambient air. The parameters evolutions within the early hours are inserted in each graph.

The surface conditions, film morphology, and carrier dynamics in CQD solids altered by ambient water would eventually determine the photovoltaic performance. Figure 4a shows the solar cell architecture and device performance. The band-gap diagram of solar cells at the working conditions and the energy level of each functional layer are presented in Fig. 4c–e and Supplementary Fig. 17. The detailed device parameters are summarized in Table 2. For the solar cells prepared under dry air, the CA devices yield a well-matched energy level alignment as shown in Fig. 4c, giving an average power conversion efficiency (PCE) of 11.2% with a remarkable FF of 69.5%. A champion PCE of 11.6% was achieved, which is so far the best reported CQD solar cells prepared by meniscus-guided-coating techniques. The fast charge transport as proved by the rapid spectra diffusion in TAS and higher electron mobility in Table 1, as well as the longest electron recombination lifetime (*τ*_*r*_, Fig. 4b, Supplementary Fig. 18) account for the efficient charge collection and thus high device FF. The spin-coated cells prepared under dry air show an average PCE of 10.2% with a moderate FF of 63.7%. The morphology defects with the significant appearance of organic residuals in CQD solids may harm the charge collection and device FF (Fig. 4c), which can be evidenced by the small geometric capacitance (*C*_geo_) and large transport resistance (*R*_tr_) from the electrochemical impedance spectroscopy (EIS) in spin-coated cells (Supplementary Fig. 19, Table S6)^62^.

**Table 2:** Summarized photovoltaic parameters for the CQD solar cells prepared under different conditions.

Deposition condition	*V*_oc_	*J*_sc_^c^	FF	PCE^d^	*n*_ideal_^e^	*n*_min_^f^ (cm^−3^)	*V*_bi_^f^ (V)	*τ*_*r*_^g^ (μs)
Spin								
Dry^a^	0.62 ± 0.02	25.8 ± 0.92	63.7 ± 1.33	10.2 ± 0.46	1.55	1.07 × 10^16^	0.67	3.87
Humid^b^	0.57 ± 0.03	23.4 ± 2.38	39.0 ± 1.57	5.2 ± 1.27	1.84	2.08 × 10^15^	0.59	1.80
CA								
Dry^a^	0.62 ± 0.01	25.9 ± 0.47	69.5 ± 0.75	11.2 ± 0.22	1.35	3.39 × 10^16^	0.68	5.05
Humid^b^	0.60 ± 0.02	25.7 ± 1.25	64.7 ± 1.27	10.0 ± 0.49	1.62	1.02 × 10^16^	0.63	3.13

^a^The devices were prepared under a humidity-controllable glove box. The dry air is under an RH < 10%.

^b^The humid air is under an RH ~50–60%.

^c^The integrated *J*_*sc*_ is shown in Supplementary Fig. 22.

^d^Average results are based on 16 devices for each condition.

^e^The ideality factors (*n*_ideal_) are linear-fitted from the light intensity-dependent *V*_oc_ graph in Supplementary Fig. 20.

^f^The minimum charge density (*n*_min_) and build-in potential (*V*_bi_) are calculated from the Mott-Schottky plot in Supplementary Fig. 21.

^g^The recombination lifetime measured under 500 nm LED illumination of 100 mW·cm^−2^, as illustrated in Supplementary Fig. 18.

Under humid conditions, serious CQD fusion causes inter-band traps and inhomogeneous energetic landscape, limiting the charge transport and *V*_*oc*_ in solar cells. Meanwhile, the surface hydroxylation reduces the total surface dipole moment, which shifts the band edge of PbS-I CQD layer up to the vacuum level and weakens the electron blocking ability of PbS-EDT layer^63^. The change of energy level is consistent with the DFT simulation in Supplementary Fig. 2d. As a result, increased interface recombination occurs as illustrated in Fig. 4e, reflected by the reduced *τ*_*r*_ and the increased ideality factors (*n*_ideal_) (Supplementary Fig. 20, Table 2) for devices prepared under humid air. In addition, the Fermi-level shifts down in spin-coated CQD films under humid air, which leads to a lower built-in potential (*V*_bi_) (Supplementary Fig. 21, Table 2). Resultantly, the humid ambient results in inferior device performance for the spin-coated devices, due to fusion-induced inter-band traps and mismatched energy levels in solar cells. The average PCE drastically drops from 10.2% to 5.2% with the FF reduced from 63.7 to 39.0%. The adverse effects of ambient water on the CQD devices prepared by different CQD inks are also investigated in Supplementary Fig. 22g–i. Fortunately, benefiting from the self-draining effect, the photovoltaic performance of CA devices is much less affected by humid air, still offering a decent PCE of 10.0%.

To exam, the possible influence of ambient water on device stability, the aging of CQD solar cells accelerated by continuous heating at 85 °C under humid ambient (RH ~50–60%) is monitored in Fig. 4f. The devices exhibit fast gains in PCE within several minutes^64^, followed by continuous decays in FF and PCE during the subsequent heating period. CA devices exhibit apparently better thermal stability than spin-coated ones. However, the decay is still significantly faster than the devices stored at room temperature. To elucidate that, the XPS of PbS-I CQD film exposed to humid air under continuous heating is collected and shown in Supplementary Fig. 6b–d. The long-time surface hydroxylation massively shrinks the iodine coverage on CQD surfaces, following the reaction in Eq. 4. In addition to the serious surface I loss, the byproduct of I_2_ gas may lead to significant iodine migrations in CQD devices, especially in spin-coated ones, as shown in the STEM-EDX images (Supplementary Fig. 23). The I migration could damage the buffer layers and electrodes, harming the charge extraction reflected by incessantly dropped FF and *J*_sc_ in solar cells^65^. Moreover, the enhanced fusion with continuous heating under humid air will inevitably cause an uneven energetic landscape, eventually reducing device performance and stability. It is worth mentioning that protecting CQD devices from the ambient humidity by device encapsulation can decelerate the decay, while the entrapped water inside the film, as evidenced by the enhanced O1*s* signals in Supplementary Fig. 5c, will still lead to device degradation under annealing (Supplementary Fig. 24). In short, we attribute the performance decay under humid ambient with annealing to the iodine loss and migration during the hydroxylation process, as well as related aggravation of CQD fusion. This revealed effect of ambient water may provide a clearer clue for the understanding of the experimental observations on trap behaviors, device deficiency, and instability in lead chalcogenide CQD electronics involving hydroxyl issue^16,17,19,21,23,66–68^.

## Discussion

In summary, we identified that the hydroxylated PbS CQD surfaces are covered by H-bonded water, which governs the temperature-dependent evolution of the surface chemical environment and generates significant effects on CQD nanostructure morphology, optoelectronic properties, as well as final photovoltaic performance. The entrapped water in CQD solids enhances the surface hydroxylation, aiding the fusion of neighboring CQDs in spin-coated film. Consequently, the disordered nanostructure morphology leads to inhomogeneous energetic landscapes and inter-band trap states, resulting in significantly broadened Stokes shift and reduced device performance. Moreover, iodine loss and migration during the hydroxylation process were revealed as the key factors for the poor thermal stability of PbS CQD solar cells. To address these issues, convective assembly was utilized for the fabrication of CQD solids. The improved homogeneity and compactness in CQD films introduce a self-draining effect to alleviate ambient water intrusion, leading to suppressed surface hydroxylation and fusion. Our result will shed light on the fundamental understanding of water-involved CQD surface chemistry and its effect on CQDs properties and photovoltaic performance, which may generate a far-reaching influence on the ambient scalable fabrication of CQD based electronics.

## Methods

### Synthesis of PbS CQDs

All operations were performed under a nitrogen atmosphere using standard air-free Schlenk line techniques. For the synthesis of ~3 nm PbS CQDs. A solution of 380 mg of lead acetate trihydrate (1 mmol), 0.7 g of oleic acid (2.5 mmol), and 20 g of 1-Octadecene (ODE) was degassed at 100 °C in a 100 ml three-neck flask for 1 h under vacuum. The solution was then heated for an additional 1 h to 150 °C under nitrogen. After adjusting the solution to the desired temperature, 0.5 mmol hexamethyldisilathiane ((TMS)_2_S) dissolved in 5 ml ODE was rapidly injected into this hot solution. The CQDs were grown at 80 °C for the optimal time, and the reaction was rapidly quenched by placing the flask in a room-temperature water bath and injecting 8 ml of anhydrous hexane, then purified by precipitation in hexane and isopropyl alcohol and once in hexane/acetone and stored with solid form in a nitrogen-filled glove box.

### Photoemission spectroscopy (PES)

Ultra-violet photoelectron spectroscopy (UPS) and X-ray photoelectron spectroscopy (XPS) were carried out in an ultra-high vacuum (UHV) system with a base pressure of 1.0 × 10^−10^ mbar. To avoid water adsorption during the transfer from the glove box to UHV, the samples were transferred in a transfer box filled with ultra-pure N_2_ gas to the load-lock chamber of the UHV system with a base pressure of 1.0 × 10^−7^ mbar. XPS was measured with a monochromatized Al Kα light source (1486.75 eV), while for UPS the likewise He Iα line (21.22 eV) was used.

### X-ray absorption spectroscopy (XAS)

The O K-edge XAS spectra were measured at beamline 20A1 of Taiwan. The PbS CQD samples were prepared on top of a silicon wafer under different conditions. The commercial Pb(OH)_2_, PbO powders were compressed into films on top of Au wafers by a tablet machine. All samples were transferred under the N_2_ box. Before measurements, the samples were first mounted onto the sample holders in a special glovebox attached to the load-lock chamber and then transferred to the analysis chamber under ultrahigh vacuum conditions. The O K-edge XAS spectra in total-electron-yield (TEY) mode was first normalized to the beam flux measured by a gold mesh and then normalized to the absorption pre-edge and post-edge. The photon energy was calibrated by the reference O K-edge spectra of SrTiO_3_.

### Density functional theory (DFT)

All density functional calculations were performed by using VASP code^69^. The electronic structures of the system were calculated with the Perdew-Burke-Ernzerhof revised for solids (PBEsol) functional and the projector-augmented wave (PAW) method under cut-off energy of 800 eV. The (5d)^10^ (5 s)^2^ (6 s)^2^ (6p)^2^ valence electrons of lead, (3 s)^2^ (3p)^4^ valence electrons of sulfur, (5 s)^2^ (5p)^5^ valence electrons of iodine, (2 s)^2^ (2p)^4^ valence electrons of oxygen and (1 s)^1^ electron of hydrogen were considered in the calculations. 4 × 4 × 4 and 4 × 4 × 1 Γ centered *k*-points sampling of the Brillouin zone were used for optimizing both the cell parameters and the atomic coordinates of the bulk and slab structures, respectively. The optimized lattice parameters of the (111) oriented bulk Pb_12_S_12_ structure were 10.22 Å, 7.23 Å, and 8.35 Å for A, B, C, respectively. The lattice parameter of the (111) oriented slab was 100 Å for A and the B and C parameters of them were kept to those of the bulk structure. The surface energy $\gamma$ is calculated as: $\gamma =\left[E\left(\text{nPbS/mPb}{\text{L}}_{2}\right)-\text{n}E\left(\text{PbS}\right)\text{-m}E\left(\text{PbL}\right)\right]/A$, where *E* (X) is the total energy of the system X, *A* is surface area, n and m represent the numbers of PbS and PbL units in the system, respectively, and L represents the capping ligands such as iodide, OH group, and oxygen anion. The total energies of PbS and PbL were taken from bulk PbS and PbL precursor molecules, respectively. The formation energy of the surface vacancy of the radical species is calculated as (the details are summarized in Supplementary Table 2):

5 $${E}_{{{\mathrm{vac}}}}\text{=}\left[E\left({\text{Pb}}_{{x}}{\text{S}}_{{y}}{\text{L}}_{{z}}\right)\text{-}\left(E\left({\text{Pb}}_{{x}}{\text{S}}_{{y}}{\text{L}}_{{z-}2}\right)\text{+}2E\left(\text{L}\right)\right)\right]\text{/}2$$

Fermi energy term is not considered in this calculation because all Pb_*x*_S_*y*_L_z_, Pb_x_S_*y*_L_*z*−2_, and L are electrically neutral because L is radical species. The water adsorption energy is calculated as:

6 $${E}_{{{\mathrm{ad}}}}\text{=}\left[\left(E\left({\text{Pb}}_{{x}}{\text{S}}_{{y}}{\text{L}}_{{z}}\right)\text{+}2E\left({\text{H}}_{2}\text{O}\right)\right)\text{-}E\left({\text{Pb}}_{\text{x}}{\text{S}}_{{y}}{\text{L}}_{{z}}\text{+}2{\text{H}}_{2}\text{O}\right)\right]\text{/}2$$

The density of states (DOS) is shifted to align the vacuum level to zero.

### Fabrication of CQD solar cells

ZnO nanoparticles were synthesized and prepared according to the reported method^18^. The ZnO NCs solution was then spin-coated on a clean ITO substrate at 2500 rpm for 30 s. The iodine passivated CQD active layer was prepared by the layer-by-layer (LbL) method.

#### Spin coating

The solid-phase-ligand-exchanged PbS-I CQD stakes were prepared by spinning coating 100 μL PbS CQDs from 40 mg mL^−1^ hexane solution at 2500 rpm. The ligand exchange was carried out by dropwise coating the TBAI 10 mg ml^−1^ tetrabutylammonium iodide (TBAI) methanol solution for 30 s on as-prepared films, and then rising with pure methanol followed through washing with acetonitrile and drying by a nitrogen gun. This procedure was repeated 6 times to achieve a nearly 250 nm thickness. The humidity was controlled from 10 to 60% by a humidifier within the glove box. The humid condition was applied with an RH of ~50–60% unless specifically mentioned. The dry air is using compressed air gas with an RH of less than 10%.

#### Convective assembly

Ten microliters of the CQDs solution was placed between the glass blade and ITO substrate which keeping an angle of 45°. A stepper motor was used to recede the substrate with uniform velocity. Different conditions were optimized according to a microscopic model proposed by Mae¨l Le Berre et al.^70^. The optimized deposition conditions were using hexane as CQDs solvent with a concentration of 80 mg/ml and a receding speed of 1 mm s^−1^. After ligand exchange, acetonitrile was dropwise added on top of the substrate followed by quick drying through a nitrogen gun. The average thickness of each deposition cycle is around 30 nm. This procedure was repeated to achieve a nearly 250 nm thickness. After the deposition of PbS-I CQD layers, 20 mg ml^−1^ PbS deposited on top of PbS-I layers followed by an EDT solution (0.04 vol% in acetonitrile) treatment for 30 s and acetonitrile rinsing twice. This procedure was repeated twice to get a thickness of 40 nm. The prepared devices were stored under dry air overnight for further oxidation and sequentially annealed at 85 °C for 30 min before the evaporation of the gold electrode. Finally, a 100 nm Au layer (0.5 Ås^−1^) was evaporated through thermal evaporation under a high vacuum lower than 1 × 10^−5^ mbar through a shadow mask to define a total active area of 0.0725 cm^2^. The solution-phase ligand exchange of PbS CQDs and the direct synthesis of PbS CQD inks were performed according to our previous reports^18,71^.

### Measurements and characterizations

Current density–voltage (*J–V*) characteristics of the devices were measured under ambient air using a programmable Keithley 2401 source meter under a simulated AM 1.5 G solar irradiation of 100 mWcm^−2^. (Newport, Class AAA solar simulator, 94023A-U) The light intensity is calibrated by a certified Oriel Reference Cell (91150 V) and verified with an NREL calibrated Hamamatsu S1787-04 diode. A metal mask was used for defining the active area (0.725 cm^2^). Voltage swept from −0.8 V to 0.8 V with a speed of 0.01 V per point and a dwell time of 10 ms. Atomic force microscopy (AFM) images were obtained using a Veeco Multimode V instrument in tapping mode. As for the cross-section TEM and images, the thin film samples were prepared by FIB after carbon coating. The TEM was operated with JEOL JEM-2010FEF at 200 kV. To monitor the evolution of CQD nanostructures affected by ambient conditions, the CQD samples in Fig. 2a–e were prepared by dropping the CQDs octane solution (2 mg/ml) on carbon meshes. Under slow evaporation of the solvent, a single-layered CQD superlattice structure can be obtained (Fig. 2a). The carbon meshes were then dipped into ligand solutions for the ligand exchange process and put on a hot plate for further heating under dry or humid conditions. The UV–vis–NIR spectra of CQD films were recorded on a Perkin Elmer model Lambda 950 under reflection mode. The steady-state photoluminescence and time-resolved luminescence spectra were measured by exciting the PbS CQD films on quartz substrate at room temperature with a pulsed Nd: YAG laser, using the frequency-doubled line at 531 nm. The luminescence was detected with a Hamamatsu R5509-73 photomultiplier. The photoluminescence lifetimes were measured using a time-correlated single-photon counting system (Hamamatsu C7990). The transient absorption spectroscopy (TAS) was carried out on an fs-TA system. The laser source was a Ti/sapphire laser (CPA-2010, Clark-MXR Inc.) with a wavelength of 775 nm, a pulse width of 150 fs, and a repetition rate of 1 kHz. The CQD film was sealed into a transparent quartz cuvette (size: 60 mm 10 mm 40 mm) filled with N_2_ gas, and all samples were pumped by a 470 nm laser pulse. The electronic recombination lifetime of the CQD device was captured using the intensity-modulated photovoltage spectroscopy (IMVS) profiling by Zahner IM6 electrochemical workstation, applying a bias of varying intensities of light illumination under open-circuit conditions with a 500 nm LED and a frequency between 0.25 MHz and 0.05 Hz. The yielded *f*_IMVS_ is inversely proportional to the electron recombination time constant *τ*_*r*_ by:

7 $${\tau }_{r}=\frac{1}{2\pi {f}_{{\mathrm{IMVS}}}}$$

Where *τ*_*r*_ is the electronic recombination lifetime, *f*_IMVS_ is the characteristic frequencies correspond to the peaks in the imaginary part in the Bode plot (see Supplementary Fig. 18).

Space charge limited current was conducted using a device structure of ITO/ZnO/PbS-I/ZnO/Al. The electron mobility was calculated according to

8 $$J=\frac{9}{8}\cdot \varepsilon {\varepsilon }_{0}\mu \frac{{V}^{2}}{{d}^{3}}$$

Where *μ* is the charge carrier mobility, *d* is the film thickness, *ε*_*0*_ is the vacuum permittivity, while *ε* represents the material’s dielectric constant, here we used for 18.7 for PbS-I. The relation between the trap density and the onset voltage of the trap-filled-limit (TFL) regime is:

9 $${V}_{{\mathrm{TFL}}}=\frac{e{N}_{t}{d}^{2}}{2\varepsilon {\varepsilon }_{0}}$$

where *e* is the unit charge, *d* is the film thickness, and *N*_*t*_ is the trap state density.

The light-dependent open-circuit voltages were measured under a 500 nm LED with adjustable illumination from 100 mW cm^−2^ to 1 mW cm^−2^, the light ideality factor is calculated according to

10 $${n}_{L}=\frac{q}{{k}_{b}T}\cdot \frac{dV}{d({\mathrm{ln}}(L))}$$

Where *k*_*b*_ is the Boltzmann constant, *T* is the temperature, *L* is the normalized light intensity.

### The molecular dynamic simulations

are based on CQD modules with {100} and {111} surface facets in truncated octahedral shapes. The {111} facets are ended with Pb atoms and the –OH groups are attached to Pb atoms to keep the whole PbS particle in charge neutral. Molecular dynamics simulations are performed with GROMACS [*Comp. Phys. Comm*. **91**, 43–56 (1995); *SoftwareX*, **1–2**, 19–25 (2015)] software and the interactions between atoms were described by a pair potential including a short-range two-body part and a long-range Coulomb part (*Nano Lett*. **10**, 3966–3971 (2010); *Sci. Adv*. 2019, **5**, eaaw5623)

11 $${{\rm{U}}}_{ij}({r}_{ij})=\frac{{q}_{i}{q}_{j}}{4\pi {\varepsilon }_{0}{r}_{ij}}+4{\varepsilon }_{ij}[{(\frac{{\delta }_{ij}}{{r}_{ij}})}^{12}-{(\frac{{\delta }_{ij}}{{r}_{ij}})}^{6}]$$

The partial charge of Pb, S, O, and H atoms are set as 1.29e, −1.29e, −2.0e, and 1.0e, respectively. The LJ coefficients are set as *σ*_Pb _= 3.29 Å, *σ*_S _= 4.36 Å, *σ*_O _= 3.15 Å, *ε*_Pb_/*k*_B_ = 30.0 K, and *ε*_Se_/*k*_B_ = 45.3 K, *ε*_O_/*k*_B_ = 76.5 K, where *k*_B_ is the Boltzmann constant. Note that due to the lack of calculation parameters for PbS, the partial charge of S and LJ coefficient of *σ*_S_ are referred to as the parameter of Se used in MD simulation of similar octahedral PbSe CQD. For H atoms, only Coulomb interaction is considered and the LJ parameters are set to zero. The widely used TIP3P model for water is adopted [*J. Chem. Phys*. **79**, 926 (1983)]. The Lorentz-Berthelot mixing rules are used to determine the interaction parameters between different atom types.

### Reporting summary

Further information on research design is available in the Nature Research Reporting Summary linked to this article.

## Supplementary information

Supplementary Information

Solar Cells Reporting Summary

## Data Availability

All necessary data generated or analyzed during this study are included in this published article, and other auxiliary data are available from the corresponding authors upon request.
