# Supplementary material for: The effect of water on colloidal quantum dot solar cells
Source: Nat Commun. 2021 Jul 19;12:4381. doi: 10.1038/s41467-021-24614-7 (PMC8289876; doi:10.1038/s41467-021-24614-7)
Supplement: Supplementary file 1 — Supplementary Information [file 41467_2021_24614_MOESM1_ESM.pdf]

Supplementary Information

The Effect of Water on Colloidal Quantum Dot Solar Cells

Shi et.al.

Supplementary Figures

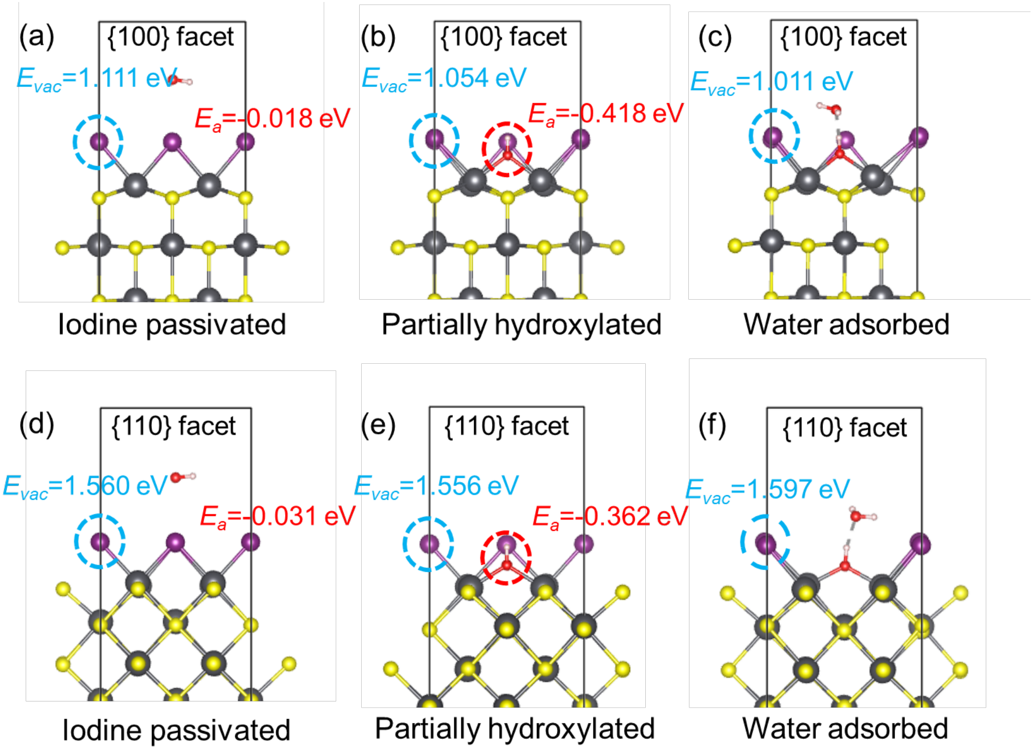

Supplementary Figure 1. The DFT calculations of E_{ad} and $E_{vac\text{-}I}$ on {100} facet and {110} facet in three different cases, including the facets covered by iodine (a, d); the hydroxylated facets (b, e) and the water adsorbed facets (c, f). The low $E_{vac\text{-}I}$ calculated on {100} and {110} facets suggest that the I adsorbed on these nonpolar facets are much more unstable than the I bonded on {111} facet.

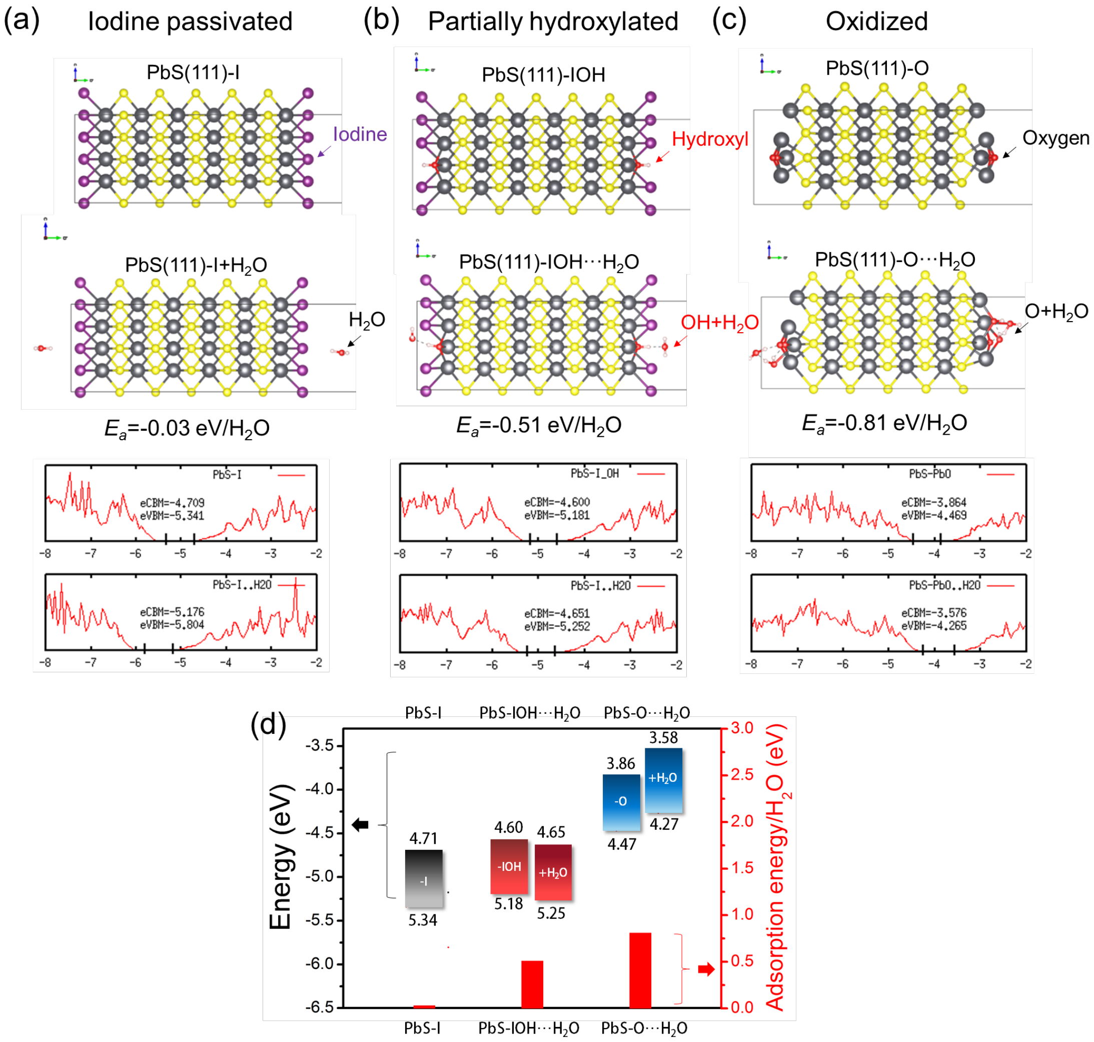

Supplementary Figure 2. The geometric structures of PbS {111} slabs with different surface conditions: (a) iodine passivated, (b) partially hydroxylated, and (c) oxidized surfaces. The corresponding electronic density of state (DOS) for each model is also shown. (d) The adsorption energy of water on PbS {111} slab passivated by different surface species (right axis). The energy levels obtained from the site-projected electronic density of state (DOS) for PbS {111} slab model capped by iodine, OH, and O, with and without water adsorption (left axis). Note that E_{CB} and E_{VB} stand for the energy level of the conduction band and valence band, respectively.

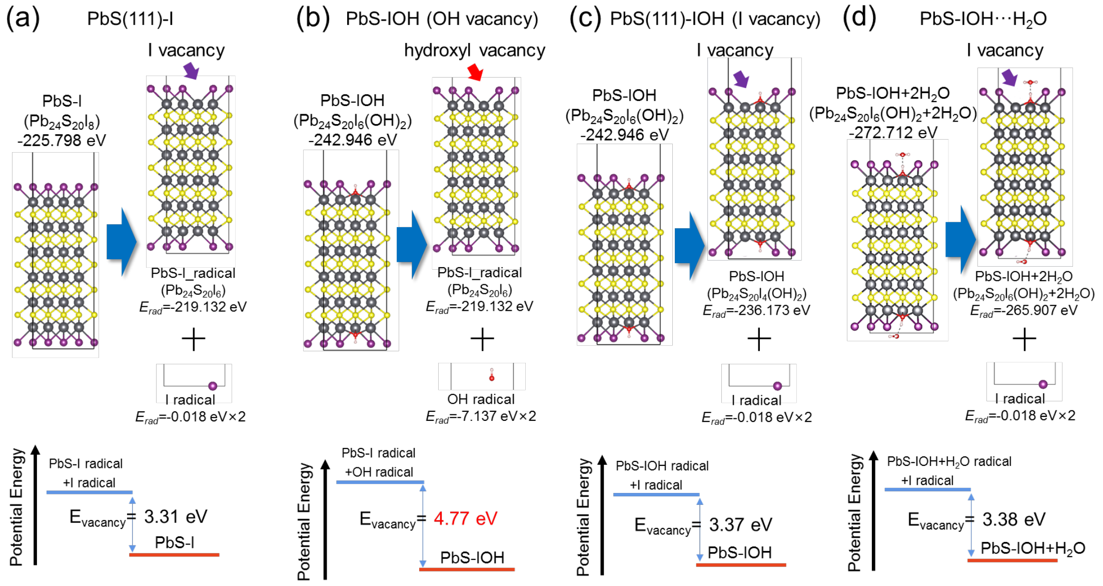

Supplementary Figure 3. The geometric structures used for the calculation of vacancy formation energy on PbS {111} facet with different situations: (a) iodine vacancy on iodine passivated surfaces. (b) hydroxyl vacancy on the partially hydroxylated surfaces. (c) iodine vacancy on the partially hydroxylated surfaces. (d) iodine vacancy on the hydroxylated surfaces stabilized by H_2O.

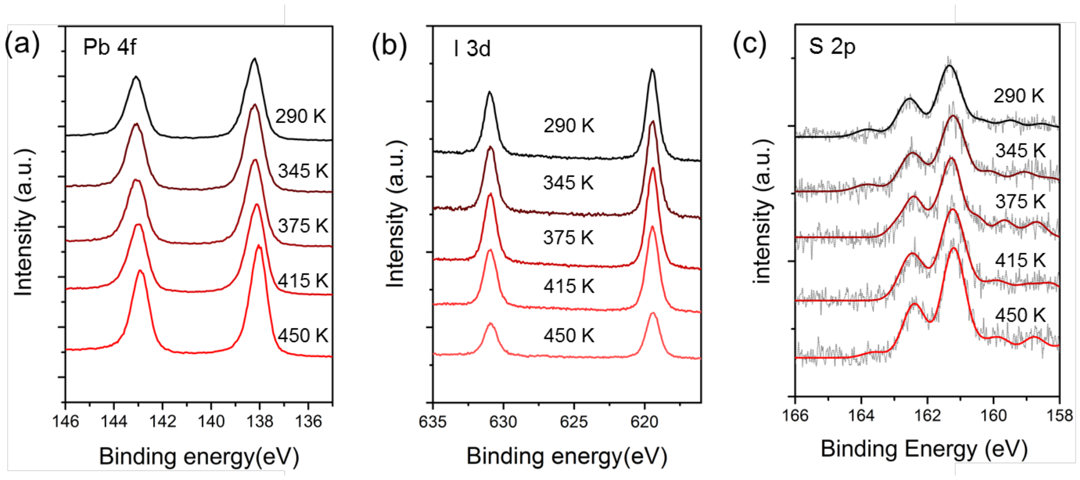

Supplementary Figure 4. Temperature-dependent XPS spectra of (a) Pb 4*f*
, (b) I 3*d*, and (c) S 2*p* of PbS-I CQD film. The atomic ratio of different surface species is summarized in Supplementary Table 1.

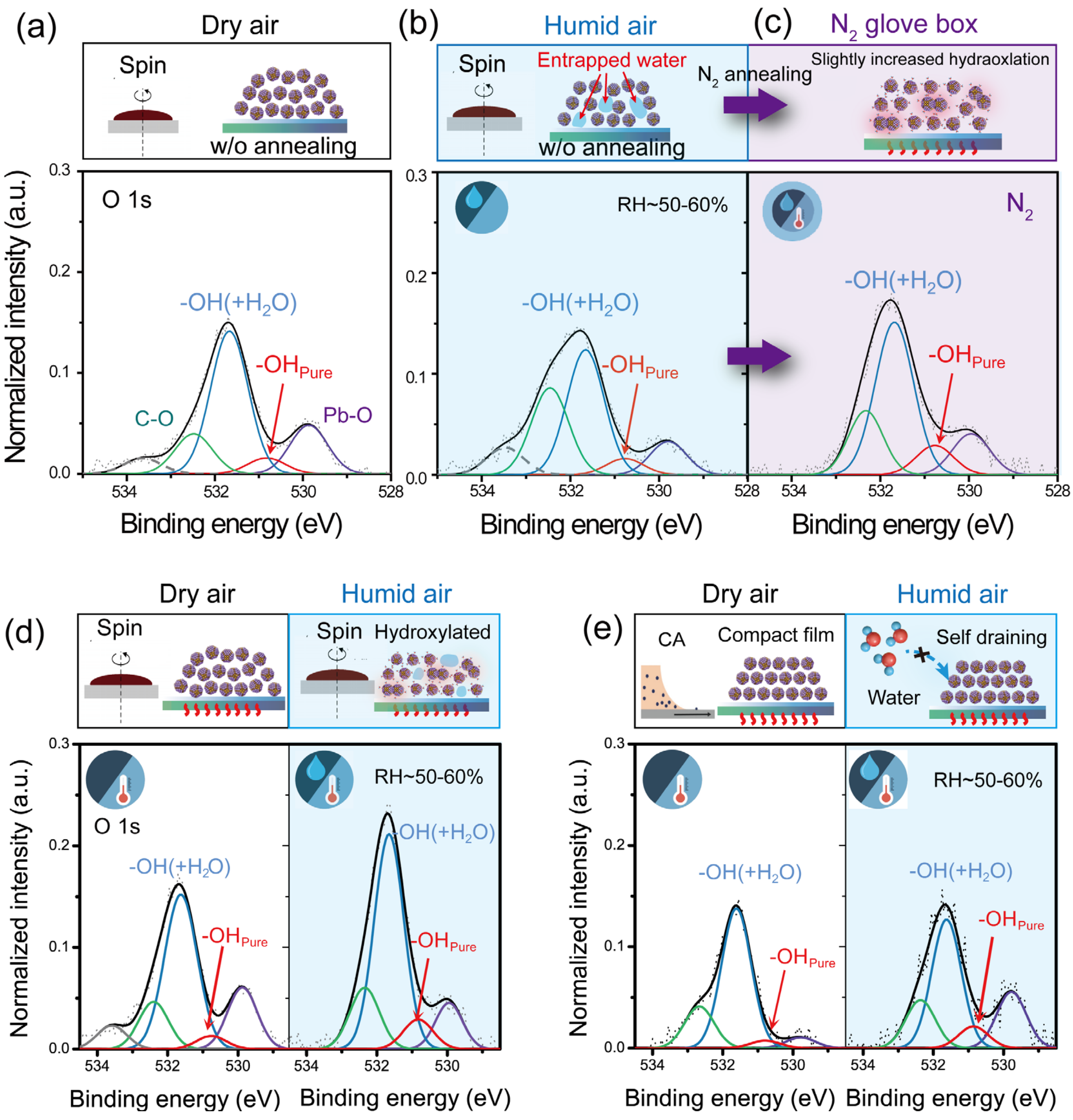

Supplementary Figure 5. O1*s* XPS spectra of PbS-I CQD films prepared under different conditions. The total peak area of all high-resolution XPS core spectra is normalized by the integrated area of each Pb 4*f* core spectra. After preparation, the samples were directly transfered from N_2 glove box to UHV chamber by a N_2 filled transfer box to avoid additional impact of ambient water on samples during delivering. Under UHV condition, most of the entrapped water should be extracted due to the relatively low H_2O-H_2O H-bonding energy of ~0.24 eV, whereas the H-bonding energy between surface hydroxyls and water molecules has a higher value of -0.51 eV per H_2O, which makes the surface water that directly H-bonded with surface OH hard to be removed even under UHV at room temperature. Thus the OH(+H_2O) phase in XPS

results under UHV mainly reflects the adsorbed water that directly H-bonded to surface hydroxyls. Note that partial surface hydroxylation already occurs during CQDs synthesis. (a) Film spin-coated under dry air and (b) humid air without annealing process. The similar peak intensity of hydroxylates between (a) and (b) indicates that annealing is necessary to increase the surface hydroxylation rapidly. (c) Film spin-coated under humid air with a subsequent annealing process at 85 °C for 30 min under N_2 atmosphere. The enhanced hydroxylation indicates that the water entrapped in CQD stacks can still contribute to the hydroxylation during the annealing process. (d) Spin-coated film under dry or humid air with a subsequent annealing process at 85 °C for 30 min. Deposition and annealing under humid air can largely increase the surface hydroxylation. (e) CA film prepared under dry or humid air with a subsequent annealing process at 85 °C for 30 min. The results suggest that humidity has less effect on CA film compared with spin-coated film.

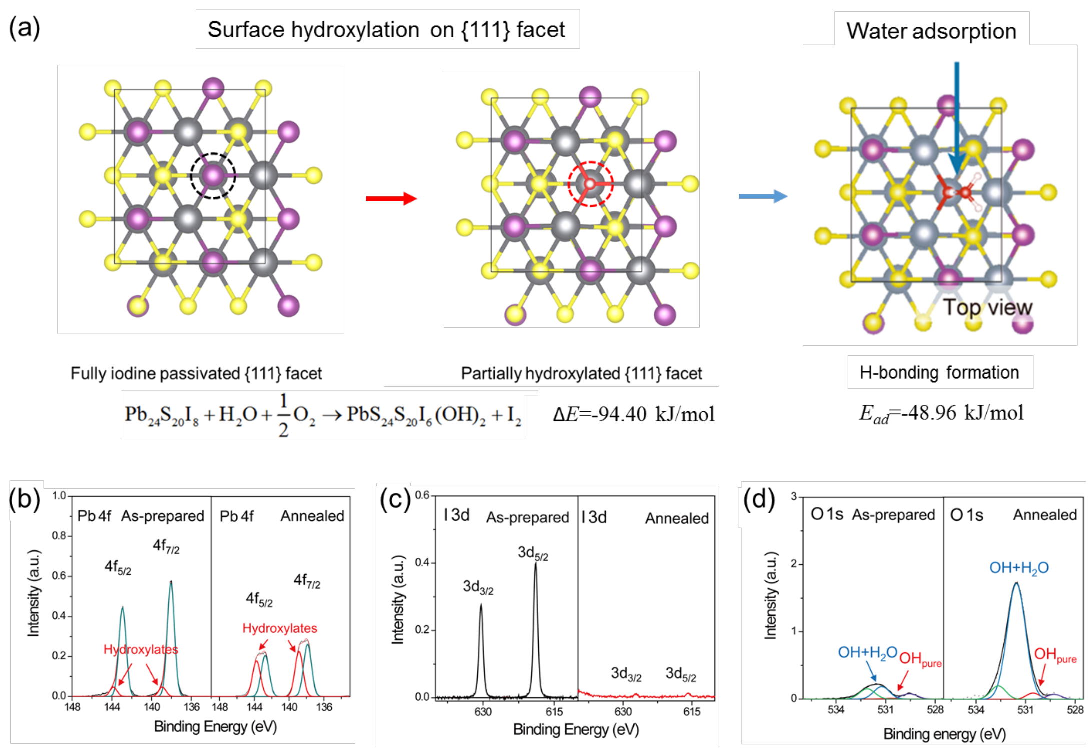

Supplementary Figure 6. (a) The hydroxylation process on PbS {111} facet under humid air are confirmed by DFT calculation. The minus ΔE=-94.40 kJ/mol suggest the reduction of free energy after surface hydroxylation on PbS {111} facet. By H-bonding formation with adsorbed water, the potential energy of the system further reduced by -48.96 kJ/mol. (b-d) XPS spectra of PbS-I films after continuous heating under ambient air (RH~40-50%) at 85 °C for one week.

Hydroxylation of PbS CQD surface during synthesis process

$$(PbS)_n(111) + Pb(OA)_2 \cdots H_2O \rightarrow OA\text{-}Pb_{n+1}S_n(111)\text{-}OH + OAH \quad \text{(S-1)}$$

$$OA\text{-}Pb_{n+1}S_n(111)\text{-}OH + H_2O \rightarrow OA\text{-}Pb_{n+1}S_n(111)\text{-}OH \cdots H_2O \quad \text{(S-2)}$$

Water chemistry on PbS-I CQD surface under UHV

Water desorption $$I\text{-}Pb_{n+1}S_n(111)\text{-}OH \cdots H_2O \xrightarrow{\Delta} I\text{-}Pb_{n+1}S_n(111)\text{-}OH + H_2O \uparrow \quad \text{(S-3)}$$

Hydroxylation $$I\text{-}Pb_{n+1}S_n(111)\text{-}I + H_2O \xrightarrow{\Delta} I\text{-}Pb_{n+1}S_n(111)\text{-}OH + HI \uparrow \quad \text{(S-4)}$$

Dehydration $$OH\text{-}Pb_{n+1}S_n(111)\text{-}OH \xrightarrow{\Delta} Pb_{n+1}S_n(111)\text{-}O + H_2O \uparrow \quad \text{(S-5)}$$

Hydroxylation and water adsorption of PbS-I CQD film under ambient air

Hydroxylation $$I\text{-}Pb_{n+1}S_n(111)\text{-}I + H_2O + \frac{1}{2}O_2 \rightarrow OH\text{-}Pb_{n+1}S_n(111)\text{-}OH + I_2 \uparrow \quad \text{(S-6)}$$

$$PbI_2(s) + H_2O(l) + \frac{1}{2}O_2(g) \rightarrow Pb(OH)_2(s) + I_2(g) \quad (\Delta G = -117.49 \text{ kJ/mol})$$

Water adsorption $$I\text{-}Pb_{n+1}S_n(111)\text{-}OH + H_2O \rightarrow I\text{-}Pb_{n+1}S_n(111)\text{-}OH \cdots H_2O \quad \text{(S-7)}$$

*The OAH stands for oleic acid. ΔG stand for Gibbs free energy.
*The (*s*) (*g*) (*l*) stand for solid, gas, and liquid, respectively.

Supplementary Figure 7. The possible chemical reaction of PbS CQDs during synthesis and heating process under UHV or humid ambient conditions.

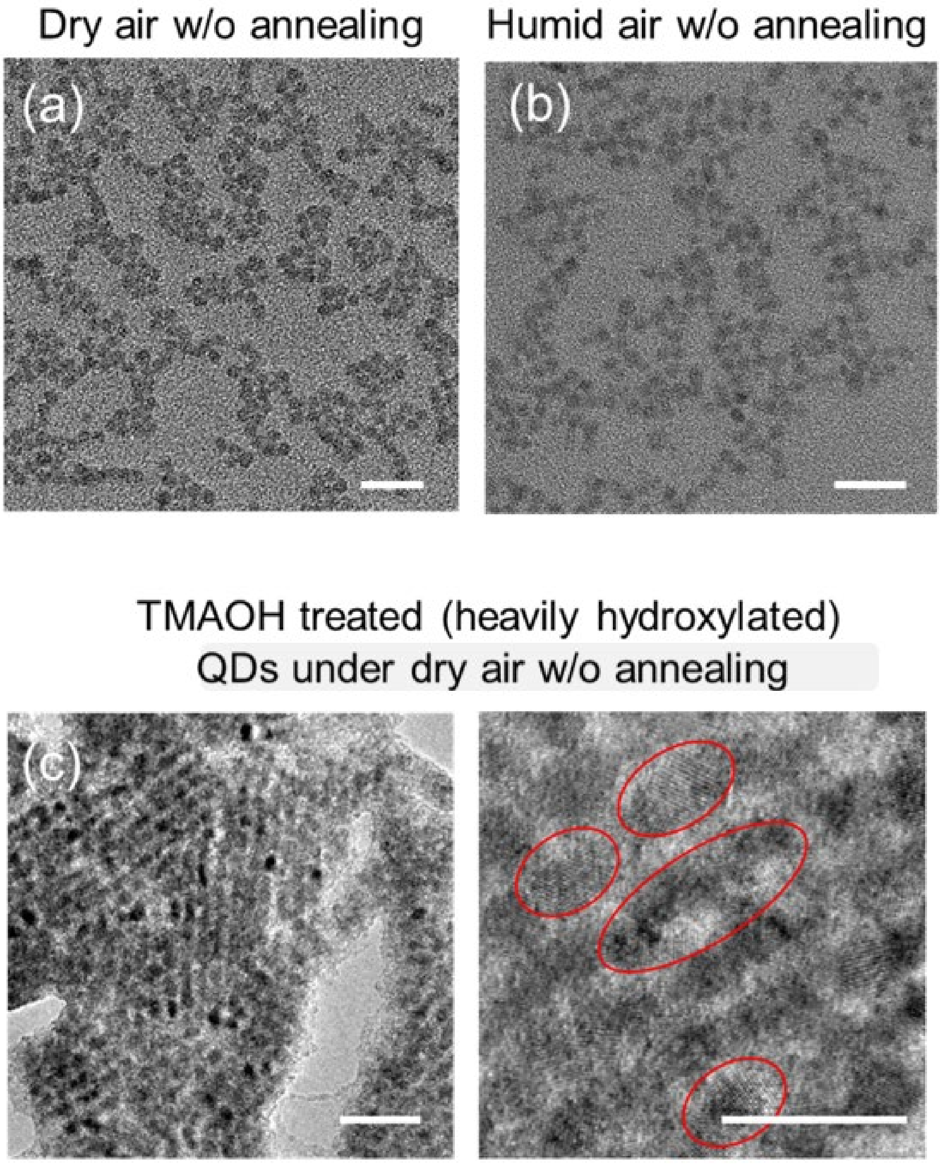

Supplementary Figure 8. TEM images of PbS-I CQDs prepared under (a) dry air and (b) humid air before annealing. (c) The TEM image of the heavily hydroxylated PbS CQDs by tetramethylammonium hydroxide pentahydrate ($TMAOH \cdot 5H_2O$) treatment in 0.1 mg/ml methanol solution for 30s. Scale bar: 10 nm. For the TMAOH treated heavily hydroxylated CQDs, oriented attachment between CQDs with serious epitaxial fusion can be found even without annealing process, which emphasizes the important role of surface hydroxylates in CQD fusion.

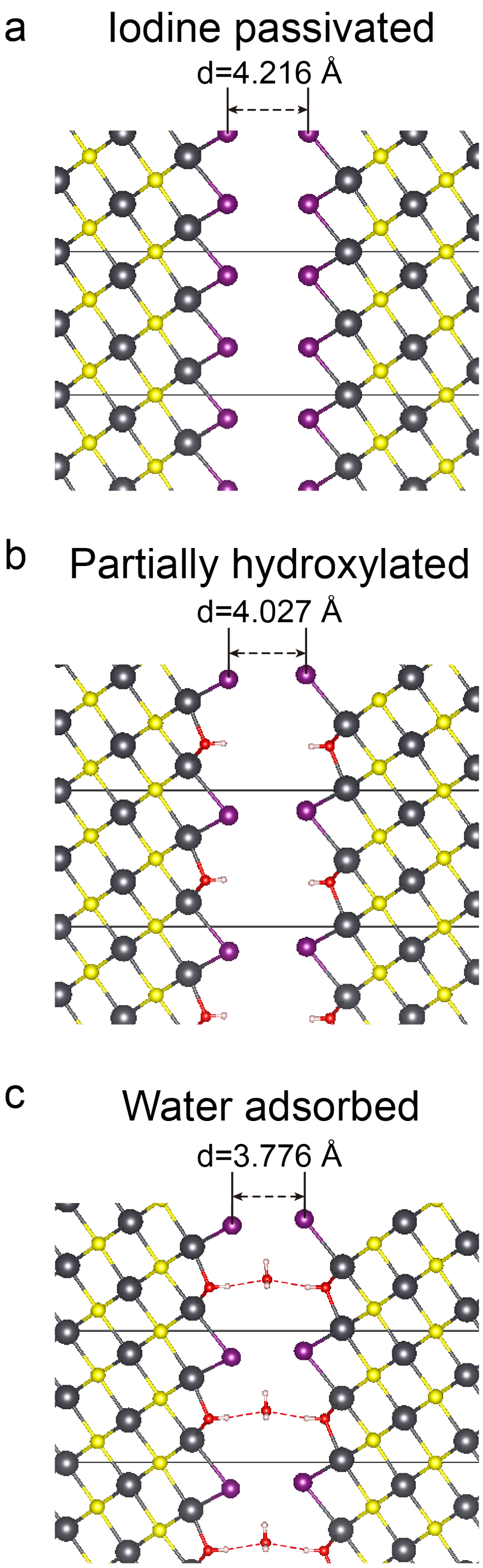

Supplementary Figure 9. DFT calculation of the relaxed inter-facet distance between two PbS {111} clusters with (a) fully iodine-passivated facets, (b) half-hydroxylated facets and (c) half-hydroxylated facets with adsorbed water. The inter-facet distance was determined by the horizontal distance between surface iodine planes on two surfaces

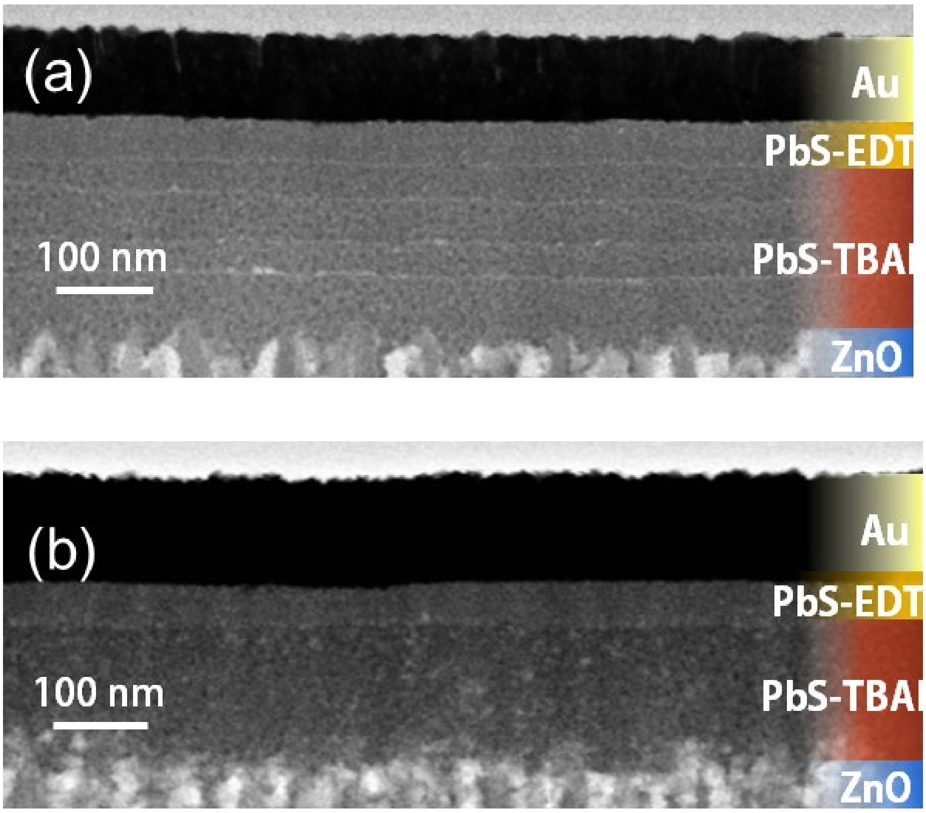

Supplementary Figure 10. (a) The cross-section bright-field FIB-TEM image of CQD stacks prepared through the LbL spin-coating method clearly shows horizontal striations inserted between each CQD layers. These stripe-like residuals visualize the low atomic number of organic components such as incompletely ligand-exchanged oleic acid (OA), TBAI residuals or voids. (b) The CA film shows compact CQD stacks without striations.

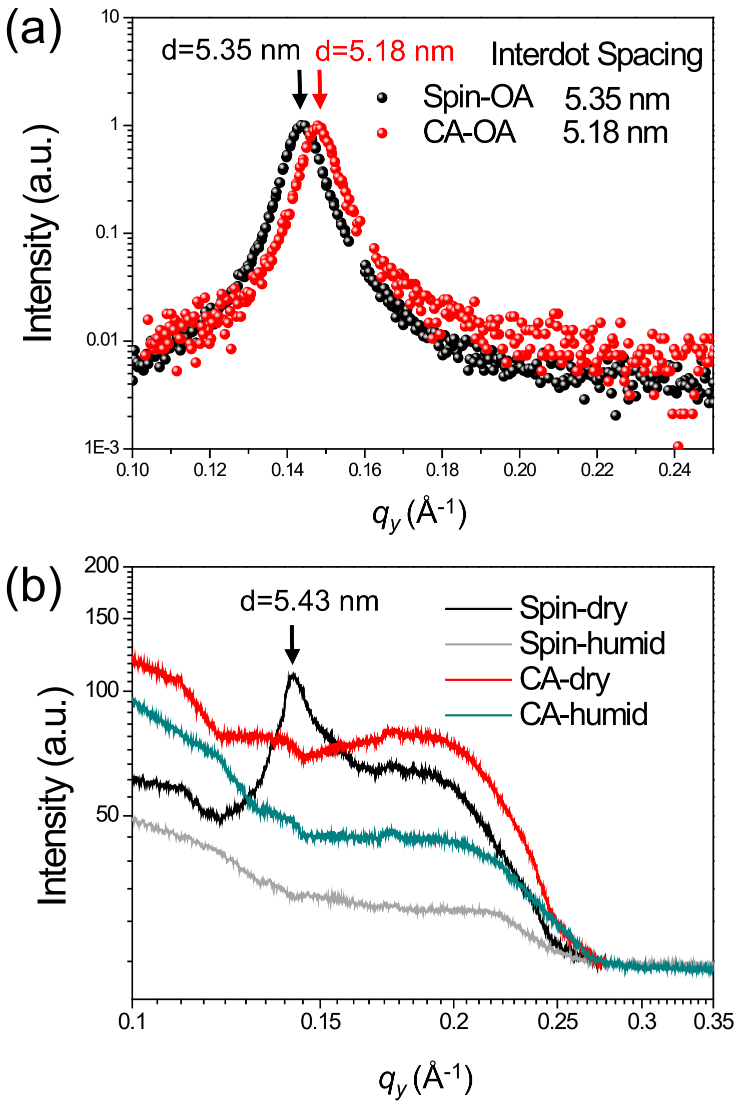

Supplementary Figure 11. (a) The cut line of the GISAXS patterns in Fig. 2g and 2k for PbS-OA CQD films before ligand exchange. (b) The azimuthally integrated GISAXS pattern of closely packed PbS-I CQD solids after ligand exchange as shown in Fig. 2h, i and l, m. The azimuthal integration is performed from 0° to 70°.

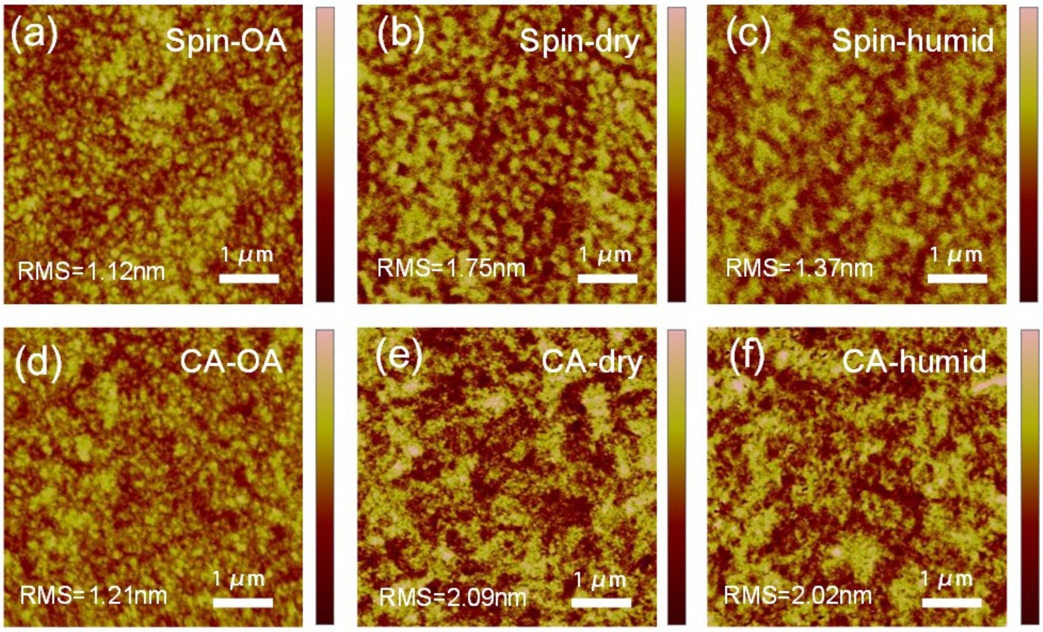

Supplementary Figure 12. AFM images of PbS CQD films deposited through (a, b, c) spin-coating and (d, e, f) convective assembly. CQD films in (a, d) and (b, e) are fabricated under dry air before and after ligand exchange, respectively. Films in (c, f) are prepared under humid air. The spin-coated CQD films show local surface aggregations, while the surface morphology of CA films is relatively more homogenous. Note that the impact of ambient humidity on surface morphology and RMS is more serious for spin-coated films, confirming the results of GISAXS.

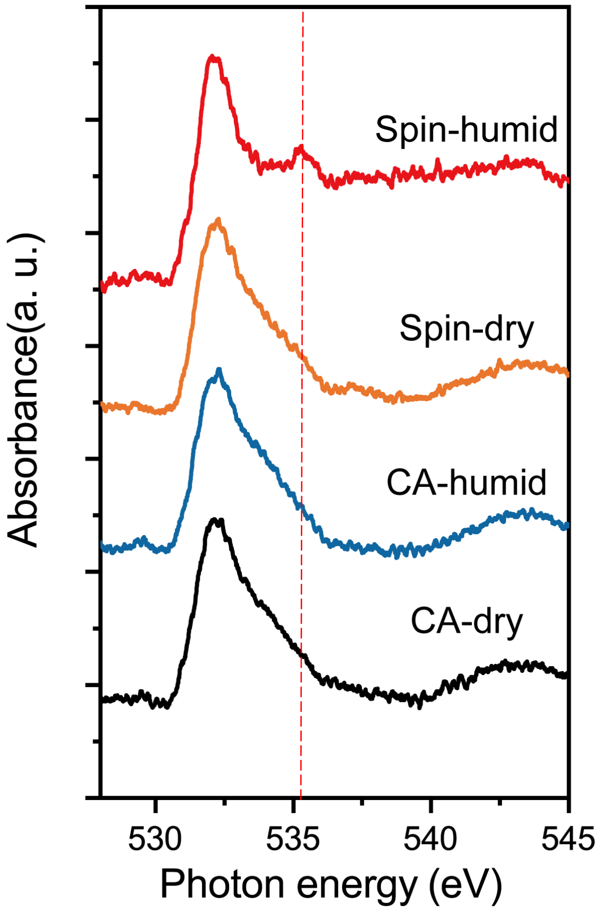

Supplementary Figure 13. Oxygen K-edge XAS spectra of PbS-I films prepared by CA under dry air (CA-dry) and humid air (CA-humid) conditions. The spin-coated curves are extracted from Fig. 1e. The similar pre-edge and main-edge XAS pattern confirm the self-draining effect of CA against ambient humidity.

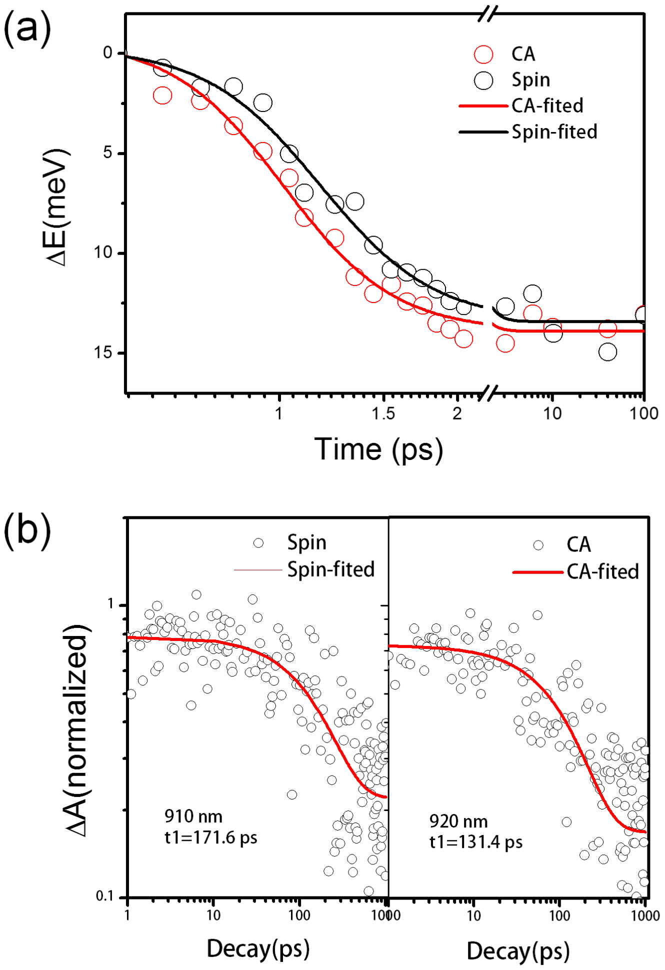

Supplementary Figure 14. (a) The shift of peak position of PIB over time for spin-coated and CA films prepared under dry air captured from Fig. 3c. The bleaching peak shifts around 13.5 meV and 13.8 meV for spin-coated and CA films prepared under dry air, respectively. The rapid shift of peak position of CA film, while with similar total energy funneling compared with that of spin-coated film, exhibits quick charge transfer due to better stacks ordering and less inter-dot spacing (enhanced QD-QD coupling). (b) Normalized TA kinetic profiles of the spin-coated and CA films prepared under dry air. The red line in spectra indicates exponential fitings. The samples were pumped by 470 nm laser pulse with a low pump flux of 6 mJ/cm^2.

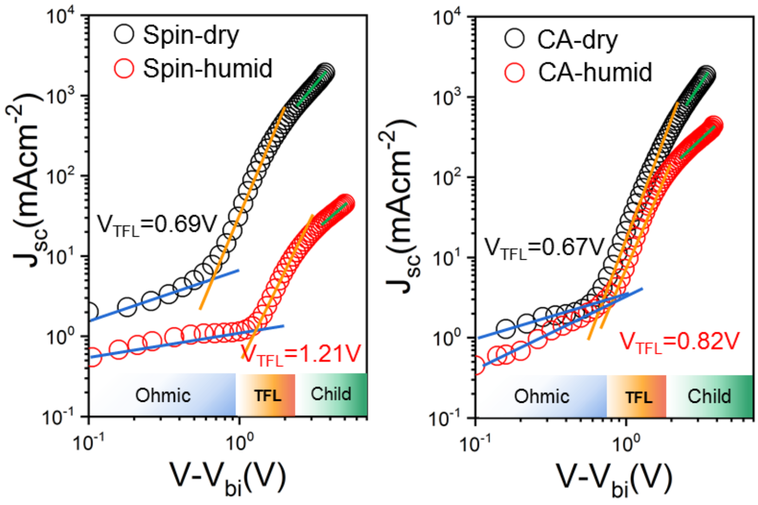

Supplementary Figure 15. Space-charge-limited current (SCLC) measurements of the electron-only devices prepared through different deposition methods. At intermediate voltages, a rapid nonlinear rise signaled transition onto the trap-filled limited region, where the trap states density (n_{trap}) is linearly proportional to the trap-filled limited voltage (V_{TFL}). The large value of V_{TFL} for spin-coated films prepared under humid air lead to high n_{trap}. A quadratic dependence of current on applied voltage can be observed in trap-free child's regime at high applied voltages. The obtained electron mobility and trap state density are presented in Table 1.

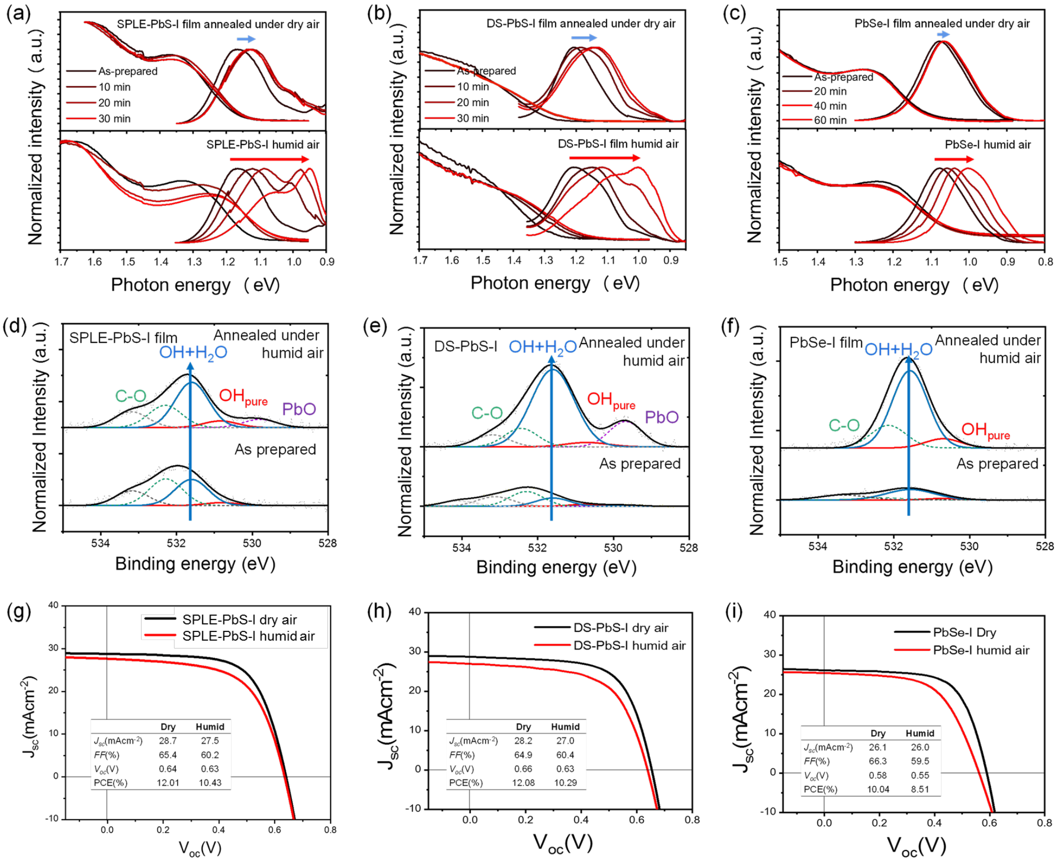

Supplementary Figure 16. The evolution of absorbance and PL spectra of (a) solution-phase ligand exchanged (SPLE) PbS-I CQD film and (b) direct-synthesized (DS) PbS-I CQD film and (c) the PbSe-I CQD films versus heating time under dry and humid air. The XPS O1*s* spectra of (d) SPLE and (e) DS PbS-I CQD films and (f) PbSe-I CQD films before and after annealing at 85 °C under humid air. The *I-V* curve of (g) SPLE and (h) DS-PbS-I CQD and (i) PbSe solar cells prepared under different ambient conditions. The insert is corresponding solar cells photovoltaic parameters. The enhanced surface hydroxylation and the broadening of Stokes shift with heating under humid air show the same trend as that of the solid-state ligand exchanged PbS-I CQD film demonstrated in Fig. 3.

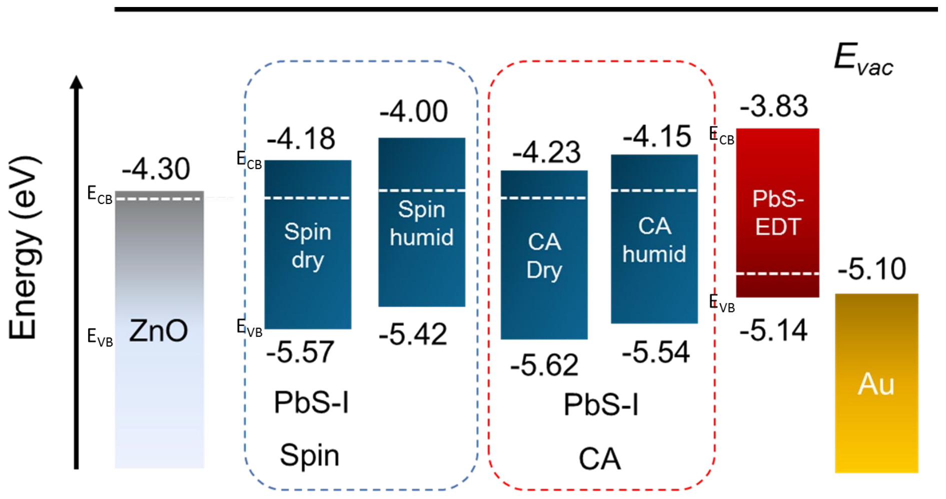

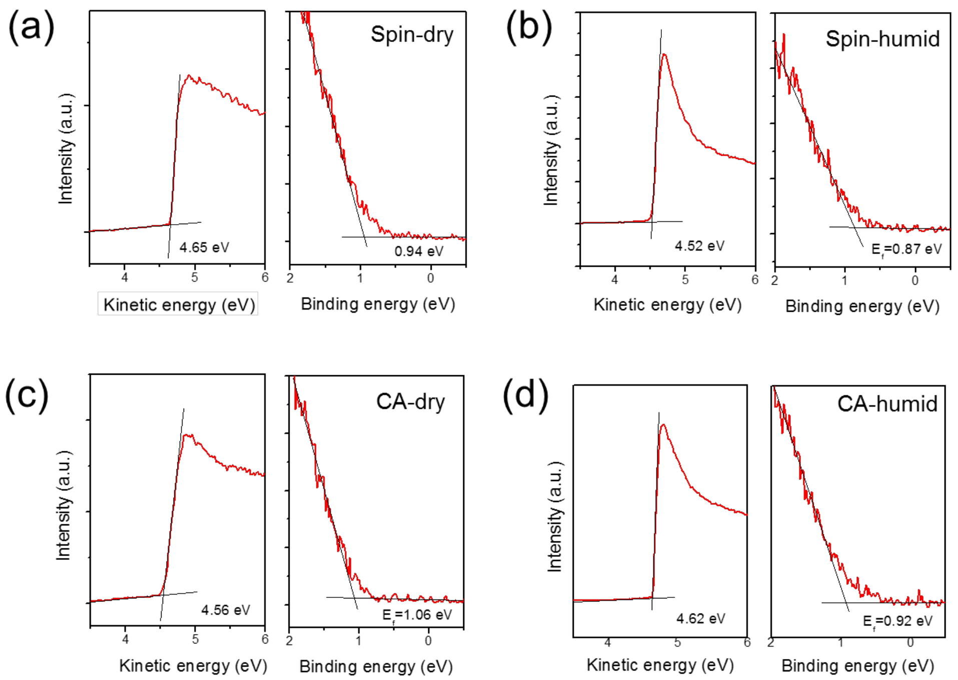

Supplementary Figure 17. Schematic illustration of the energy level alignment for PbS CQD solar cells, and the UPS spectra of PbS-I CQD films prepared by different deposition conditions. (a) The spin-coated film prepared under dry air. (b) the spin-coated film prepared under humid air. (c) the CA film prepared under dry air. (d) the spin-coated film prepared under humid air. The left panel shows the secondary electron cut-off region, and the right panel shows the magnified spectra near the Fermi edge. Note that E_{CB} and E_{VB} stand for the energy level of the conduction band and valence band, respectively.

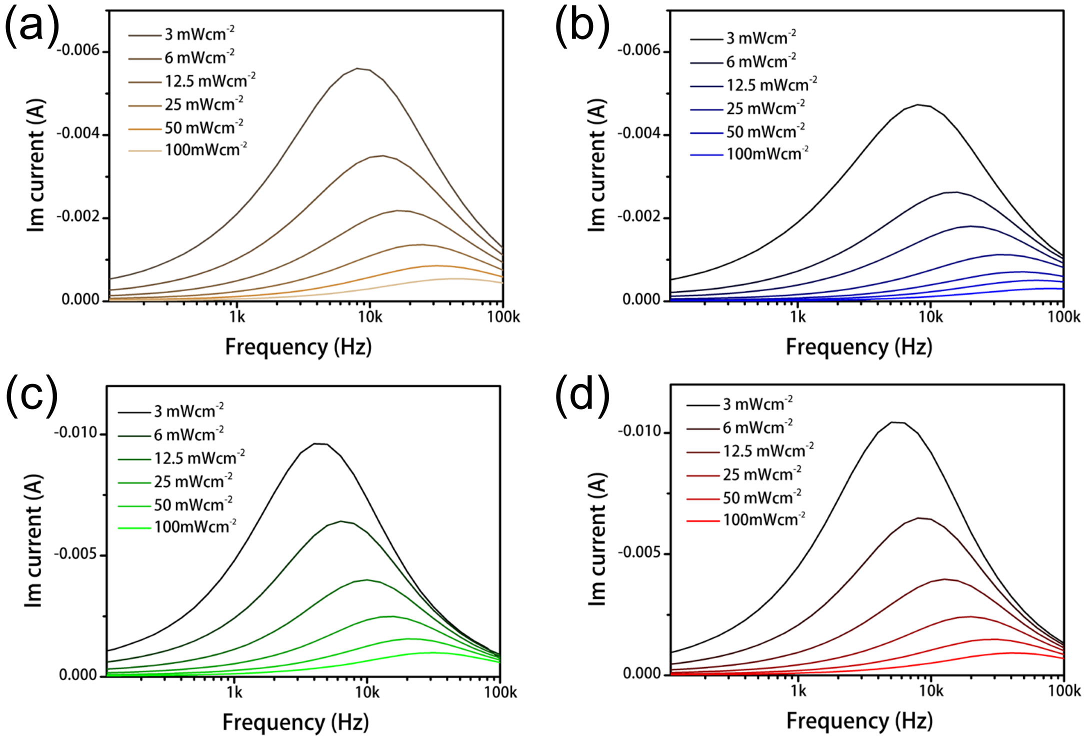

Supplementary Figure 18. Bode plot of the imaginary current part of spin-coated solar cells prepared under (a) dry and (b) humid air measured under various illumination intensities. CA devices prepared under (c) dry and (d) humid air. The IMVS represents the frequency where each imaginary part reaches its minimum value. The time constants measured under 100 mWcm^{-2} are listed in Table 1.

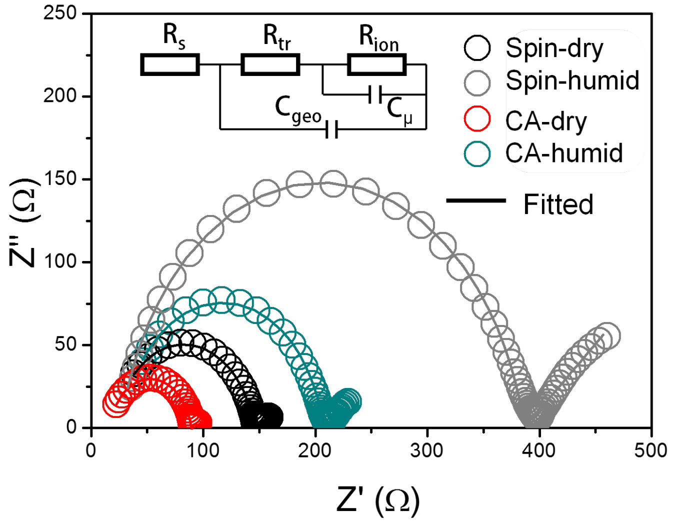

Supplementary Figure 19. Electrochemical impedance spectroscopy (EIS) of devices measured under the illumination of 500 nm LED at the open-circuit condition. The fitted series resistance R_s, transport resistance R_{tr} and geometry capacitance C_{geo} are summarized in Table S6.

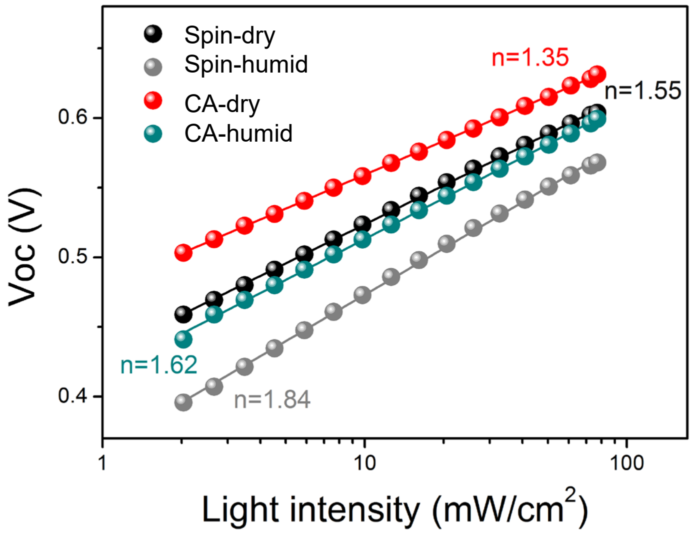

Supplementary Figure 20. Light intensity-dependent V_{oc} and calculated ideality factor of CQD devices prepared under different conditions. (solid lines: linear fits). The obtained ideality factors are listed in the graph and Table 2. A lowest n_{ideal}=1.35 can be found in CA devices prepared under dry air, demonstrating the less trap behaviour in CQD solar cells. In contrast, a high n_{ideal}=1.84 in the spin-coated device under humid air reflex serious charge recombination in solar cell.

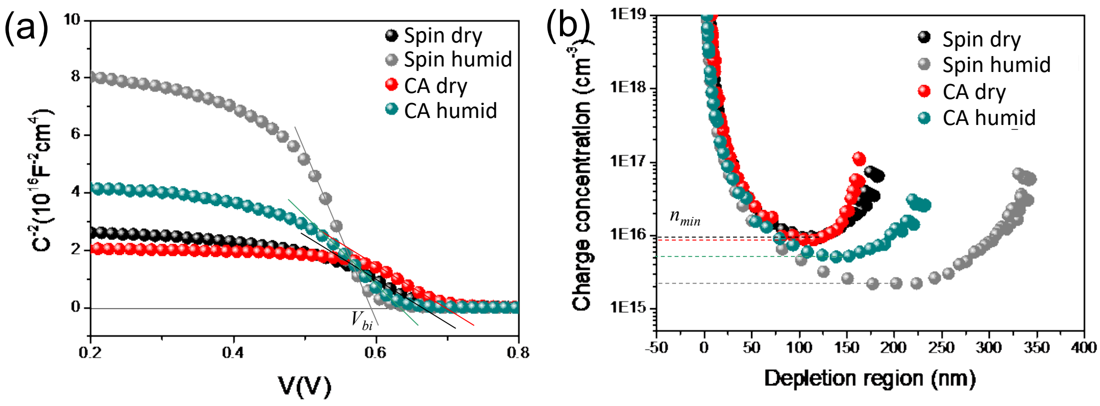

Supplementary Figure 21. (a) Mott-Schottky plot and (b) the space charge region carrier density profile obtained from (a). The obtained minimum carrier density and the depletion width are listed in Table 2. The high V_{bi}=0.67 V observed for the CA device results in sufficient driving forces for exciton separation and high V_{oc}. The spin-coated devices fabricated under humid air are suffered from the low V_{bi}=0.59V and corresponding minimum charge concentration (n_{min}) in depletion area.

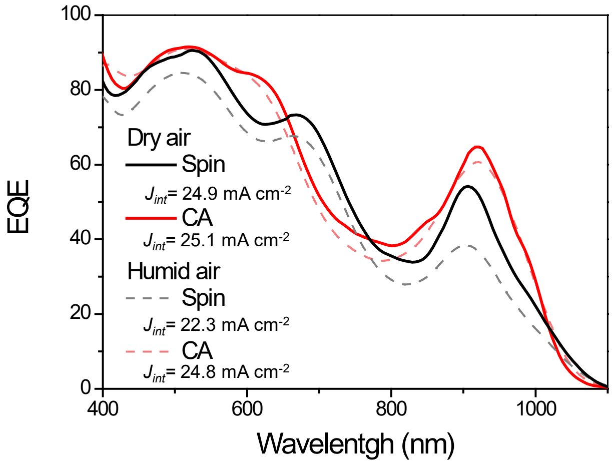

Supplementary Figure 22. External quantum efficiency (EQE) spectra of PbS CQD devices prepared under different conditions. The integrated current density for each condition is noted.

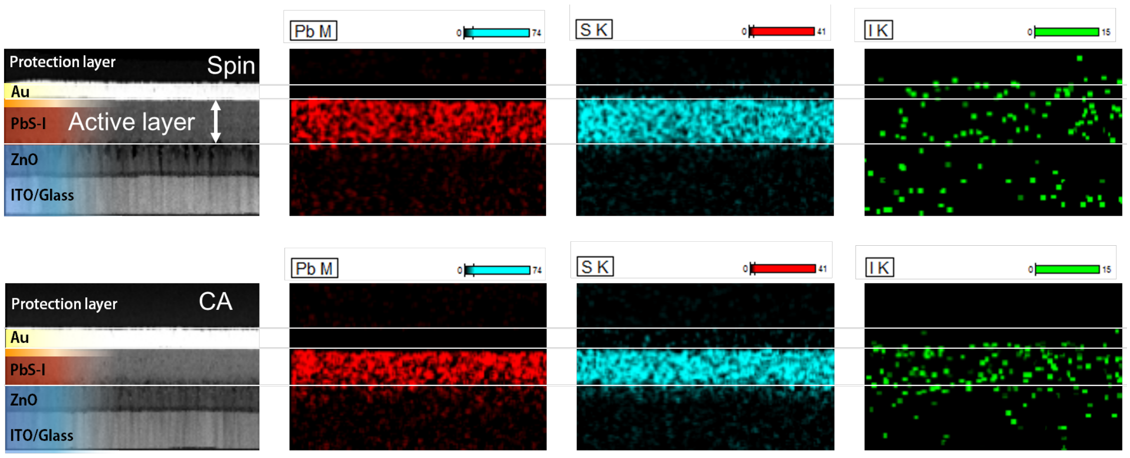

Supplementary Figure 23. Scanning transmission electron microscopy/energy dispersive X-ray analysis (STEM-EDX) mappings of PbS CQD device prepared by (a) spin coating and (b) CA. Note that the devices were annealing at 85 °C for 30 min and stored under dark at ambient air before measurement. An evident migration of iodine into gold PbS-EDT and ZnO can be concluded, especially for the spin-coated device.

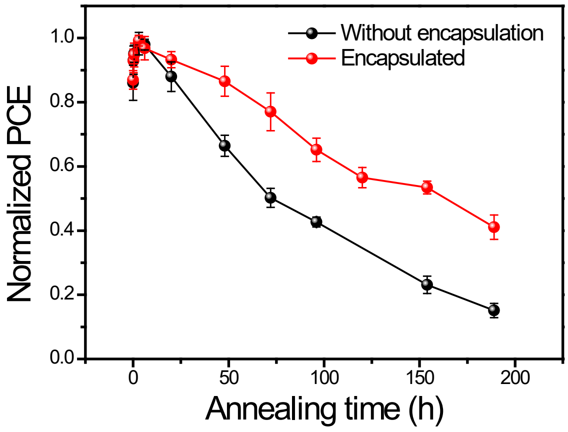

Supplementary Figure 24. Thermal stability of spin-coated CQD devices with or without encapsulation at 85 °C under ambient humid air (40-50% RH).

Supplementary Tables

Supplementary Table 1. Summary of atom distance, adsorption energy of water, and predicted conduction band minimum (eCBM) and valence band maximum (eVBM) of PbS (111) slab with different surface species including iodine passivation, partially hydroxylation, and oxidization with or without water adsorption obtained from the DFT calculation in Supplementary Fig. 2.

Surface configuration PbS(111)	Atom distance (Å)		$E_{surface\text{-}species}$ (eV) [a]	E_a (eV/H_2O) [b]	Calculated eCBM (eV)	Calculated eVBM (eV)
Pb-I	r(Pb-I)	3.18	− 225.798	-0.03	4.709	5.341
Pb-I+H_2O		3.19	− 254.605		5.176	5.804
Pb-IOH	r(Pb-I)	3.23	− 242.946	-0.51	4.600	5.181
	r(Pb-O)	2.48				
Pb-IOH···H_2O	r(Pb-I)	3.30	− 272.712		4.651	5.252
	r(Pb-O)	2.43				
Pb-O	r(Pb-O)	2.32	− 230.292	-0.81	3.864	4.469
Pb-O···H_2O		2.33	− 291.017		3.576	4.265

[a] E_{model} stands for the potential energy of different PbS(111) model before and after water adsporption.
[b] E_a stands for the adsorption energy of water on the specific surface.

Supplementary Table 2. The surface energy and vacancy formation energy of PbS {111} facet passivated with different surface species, where γ stands for the surface energy. The slab configurations are shown in Supplementary Fig. 2-3, except for the Pb-OH that is based on the slab with fully hydroxylated surfaces.

<table>
<tr><th>Surface condition PbS (111)</th><th>Surface energy (γ, J/m^2)</th><th>Vacancy type</th><th colspan="2">E_{rad} (eV)^a</th><th>$E_{passivated}$ (eV)^b</th><th>$E_{vacancy}$ /surface (eV)^c</th></tr>
<tr><td rowspan="2">PbS-I</td><td rowspan="2">0.0048</td><td rowspan="2">I vacancy</td><td>PbS-I radical</td><td>I radical</td><td rowspan="2">-225.798</td><td rowspan="2">3.31</td></tr>
<tr><td>-219.132</td><td>-0.028</td></tr>
<tr><td rowspan="4">PbS-IOH</td><td rowspan="4">-0.187</td><td rowspan="2">I vacancy</td><td>PbS-I radical</td><td>OH radical</td><td rowspan="2">-242.946</td><td rowspan="2">3.37</td></tr>
<tr><td>-219.132</td><td>-7.137</td></tr>
<tr><td rowspan="2">OH vacancy</td><td>PbS-IOH radical</td><td>I radical</td><td rowspan="2">-242.946</td><td rowspan="2">4.77</td></tr>
<tr><td>-236.173</td><td>-0.028</td></tr>
<tr><td rowspan="2">PbS-IOH···H_2O</td><td rowspan="2">-</td><td rowspan="2">I vacancy</td><td>PbS-IOH+H_2O radical</td><td>I radical</td><td rowspan="2">-272.712</td><td rowspan="2">3.38</td></tr>
<tr><td>-265.907</td><td>-0.028</td></tr>
<tr><td>PbS-OH</td><td>-5.42</td><td colspan="3">-</td><td>-</td><td>-</td></tr>
</table>

^{a} E_{rad} represents the potential energy of radicals including I, OH, and PbS(111) model with different types of surface vacancies.

^{b} $E_{passivated}$ stands for the potential energy of the PbS(111) model fully passivated by different surface species.

^{c} $E_{vacancy}$ stands for the calculated vacancy formation energy from Supplementary Fig. 3.

Supplementary Table 3. The assignment of surface species in the XPS O1*s* spectra on PbS CQD surfaces in published works.

Peak position in O1*s* spectra				Year	Reference
Pb-O	$-OH_{pure}$	$OH+H_2O$	C-O		
529.6	531.3	-	532.0	2020	[1]
-	531.1	-	532.1		[2]
528.8	531.0	-	532.4		[3]
-	531.4	-	532.2		[4]
530.3	531.1	-	531.8		[5]
-	531.3	-	532.6		[6]
-	531.4	-	532.2	2019	[7]
529.1	530.1	-	531.9		[8]
529.8	531.6	-	532.3		[9]
529.4	532.5	-	-		[10]
-	531.7	-	532.5		[11]
529.3	531.5	-	-		[12]
529.5	531.0	-	532.4	2018	[13]
529.6	531.5	-	532.5		[14]
-	531.1	-	-		[15]
530.8	531.3	-	532.0		[16]
529.5	531.3	-	-		[17]
529.3	531.2	-	532.1		[18]
529.5	531.2	-	-	2017	[19]
528.8	530.8	-	532.4		[20]
528.3	531.0	-	532.5		[21]
-	531.3	-	-		[22]
529.3	531.0	-	532.2	2014-2016	[23]
-	531.8	-	-		[24]
-	531.2	-	532.1		[25]
529.8	530.7	531.6	532.2	Our work	

Supplementary Table 4. Peak position, full width half maximum (FWHM) of surface species in O1*s* spectra, and the atomic ratio of different elements relative to Pb 4*f* core captured from the in-situ temperature-dependent XPS spectra in Fig. 1c, d and Supplementary Fig. 4. The total hydroxylates are the sum of OH_{pure} and $OH+H_2O$ species in O1*s* spectra.

Temperature	Peak position(eV)/FWHM(eV)			
	O1*s*			
	Pb-O	$-OH_{pure}$	$-OH+H_2O$	C-O
290 K	529.8/1.06	530.7/1.06	531.6/1.06	532.2/1.06
345 K	529.7/1.05	530.7/1.05	531.6/1.05	532.3/1.05
375 K	529.7/1.04	530.7/1.04	531.6/1.04	532.3/1.04
415 K	529.7/1.04	530.7/1.04	531.6/1.04	532.3/1.04
450 K	529.6/1.04	530.7/1.04	531.6/1.04	532.2/1.04

Temperature	Atomic ratio relative to Pb 4*f* core (%)							
	I 3*d*	O1*s*					Total I and O	S 2*p*
		Pb-O	OH_{pure}	$OH+H_2O$	C-O	Total hydroxylates		
290 K	54.9	6.2	1.6	19.6	8.9	21.2	81.5	72.3
345 K	62.6	7.7	2.9	15.8	3.2	18.7	89.0	67.7
375 K	66.3	6.5	4.2	13.7	4.0	17.9	90.7	72.7
415 K	64.4	5.2	6.7	11.7	3.4	18.4	85.8	75.4
450 K	22.7	12.6	9.1	5.5	1.6	14.6	55.4	77.6

Supplementary Table 5. The atomic ratio of different surface species relative to Pb 4*f* core for PbS-I films prepared under different conditions.

Deposition	Ambient humidity	Annealing process	Atomic ratio relative to Pb 4*f* core					
			I 3*d*	O 1*s*				S 2*p*
				OH_{pure}	OH ($+H_2O$)	Pb-O	C-O	
spin	low	w/o	55.3	1.7	16.2	5.5	7.2	70.4
	high		52.7	1.7	13.1	3.4	11.9	73.0
	low	Air	55.1	1.6	17.7	6.3	7.3	74.5
	high		53.9	3.1	22.5	4.4	6.4	76.1
	high	N_2	52.1	3.2	16.5	4.3	6.2	75.8
CA	low	Air	58.5	0.8	14.5	1.1	4.3	68.8
	high		59.1	2.3	14.6	5.8	5.0	71.9

Supplementary Table 6. The fitting results from EIS measurements in Figure S19. The fitted series resistance R_s, transport resistance R_{tr} and geometry capacitance C_{geo} are summarized.

Deposition Condition		R_s (Ω)	R_{tr} (Ω)	C_{geo} (nF)
Spin	Dry	16.4	126.0	105.0
	Humid	38.0	258.9	108.8
CA	Dry	19.9	73.8	197.2
	Humid	28.5	178.0	119.8

Supplementary Note 1. Explanation of the TAS in manuscript Fig. 3

The importance of CQD stack ordering and surface conditions on the inter-dot carrier transport is underlined by the transient absorption spectroscopy (TAS), as shown in Fig. 3c and Supplementary Fig. 14. The time-dependent redshift and the narrowing of the photo-induced bleaching (PIB) ($\Delta A<0$) in TAS spectra, known as the spectra funneling effect, have been suggested to be directly related to the mobility of charge carriers in CQD solids.[26, 27] The spatial transport of photocarriers accompanied by ultra-fast relaxation to the shallow state lead to the rapid spectra diffusion in TAS. In the case of PbS CQD solids prepared under dry air, the approximate total energy shifts in spin-coated and CA films reflect the similar band tail features, whereas the densely packed CA film demonstrates a much faster processing rate of spectra diffusion within several picoseconds (Supplementary Fig. 14b). A non-defect-dominated feature of decay in bleaching signal can be assured by a single-exponential fitting conducted for the TAS time traces with a low excitation density (Supplementary Fig. 14a).[28-30] Thus, the rapid spectra diffusion observed in CA film can be attributed to the fast charge transfer between neighboring CQDs due to enhanced electron coupling and flatten energetic landscape compared to the spin-coated film with multi-featured morphology.[26] The inhomogeneous CQD distribution in spin-coated film could hinder the carrier transport, which reduces the electron mobility for spin-coated films as presented in Supplementary Fig. 15 and Table 1.

Supplementary Note 2. Explanation of the origination of Stokes shift in Fig. 3a, b

Generally, the Stokes shift for CQD films can be affected by inter-dot spacing, morphological homogeneity, and trap behavior of charge carriers in CQD solids.[31, 32] For densely packed CA film with uniform morphology, as proved by the TEM and GISAXS measurement in Fig. 2l, n, the energetic landscape is homogeneous, resulting in narrow Stokes shift. For spin-coated film, the inferior morphology causes energetic disorder and water entrapping. The significant change in surface condition with enhanced surface hydroxylation may alter the effective size of CQDs, increassing stokes shift to tens of meV. [33] However, the effect is not significant enough to interpret the large Stokes shift of 305 meV for the spin-coated CQD film under humid air. Generally, the inter-band trap states play a critical role since they were reported to vary the Stokes shift to more than hundreds of meV.[34, 35] The density of Schottky vacancy on a specific facet can be determined by the vacancy formation energy (E_{vac}) at a given temperature.[36] We thus try to calculate the E_{vac} in our case. As shown in Supplementary Fig. 3 and Supplementary Table 2, the strong electron-withdrawing ability of the oxygen atom and the small steric hindrance of OH could increase the vacancy formation energy from 3.31 eV ($E_{vac\text{-}I}$) to 4.77 eV ($E_{vac\text{-}OH}$), which makes the surface OH vacancies thermodynamically hard to be formed. In Fig. 1b, for the iodine located at the neighbor site of OH+H_2O, the bonding length between Pb and iodine increases from 3.18 Å to 3.30 Å (Table 1). However, the Pb connected to the OH group shoud be more positively charged, which may offset the effect of elongated bonding length, consequently balancing the Coulomb interactions and leading to a comparable $E_{vac\text{-}I}$ of 3.38 eV. Much smaller $E_{vac\text{-}I}$ on {100} and {110} facet can be found, suggesting unstable condition of iodine on these facets. More specific passivation strategies should be used for the large size PbS CQDs or PbSe CQDs, which have more surface {100} or {110} facets. However, no significant change in $E_{vac\text{-}I}$ can be predicted between the iodine passivated and partially hydroxylated or water adsorbed {100} or {110} facets, which suggest that the introduction of surface hydroxylates could not directly increase surface vacancies. Therefore, the reasonable answer for the origination of traps in PbS CQD

solids related to surface hydroyxlates should be the epitaxial fusion of CQDs, as we observed in Fig. 2 c-e. The strong electronic coupling between two fused CQDs generates new states lower in energy by 100–200 meV relative to the single CQD bandgap, creating the inter-band traps and broadening the Stokes shift.[37, 38]

Supplementary References

1. Yang X, *et al.* Hydroiodic acid additive enhanced the performance and stability of PbS-QDs solar cells via suppressing hydroxyl ligand. *Nano-Micro Lett.* **12**, 37 (2020).
2. Sun B, *et al.* Ligand-assisted reconstruction of colloidal quantum dots decreases trap state density. *Nano Lett.*, **20**, 3694-3702 (2020).
3. Ding C, *et al.* Passivation strategy of reducing both electron and hole trap states for achieving high-efficiency PbS quantum-dot solar cells with power conversion efficiency over 12%. *ACS Energy Lett.*, 3224-3236 (2020).
4. Xia Y, *et al.* Facet control for trap‐state suppression in colloidal quantum dot solids. *Adv. Funct. Mater.* **30**, 2000594 (2020).
5. Yang J, *et al.* Hybrid surface passivation for retrieving charge collection efficiency of colloidal quantum dot photovoltaics. *ACS Appl. Mater. Inter.* **12**, 43576-43585 (2020).
6. Song JH, Kim T, Park T, Jeong S. Suppression of hydroxylation on the surface of colloidal quantum dots to enhance the open-circuit voltage of photovoltaics. *J. Mater. Chem. A.* **8**, 4844-4849 (2020).
7. Wang L, *et al.* Manipulation of phase-transfer ligand-exchange dynamics of PbS quantum dots for efficient infrared photovoltaics. *J. Phys. Chem. C.* **123**, 30137-30144 (2019).
8. Sliz R, *et al.* Stable colloidal quantum dot inks enable inkjet-printed high-sensitivity infrared photodetectors. *ACS Nano.* **13**, 11988-11995 (2019).
9. Hu L, *et al.* Synergistic effect of electron transport layer and colloidal quantum dot solid enable PbSe quantum dot solar cell achieving over 10 % efficiency. *Nano Energy.* **64**, 103922 (2019).
10. Tulsani SR, Ganguly S, Rath AK. Inorganic metal iodide mediated solution phase surface passivation for quantum dot solar cell. *J. Mater. Sci.: Mater. Electron.* **30**, 16234-16243 (2019).
11. Gu M, *et al.* Stable PbS quantum dot ink for efficient solar cells by solution-phase ligand engineering. *J. Mater. Chem. A.* **7**, 15951-15959 (2019).
12. Ahmad W, *et al.* Lead selenide (PbSe) colloidal quantum dot solar cells with >10% efficiency. *Adv Mater.* **31**, e1900593 (2019).
13. Wang Y, *et al.* In-situ passivation for efficient PbS quantum dot solar cells by precursor engineering. *Adv. Mater.* **30**, 1704871 (2018).
14. Lu K, *et al.* High-efficiency PbS quantum-dot solar cells with greatly simplified fabrication processing via "solvent-curing". *Adv. Mater.* **30**, e1707572 (2018).
15. Jo JW, *et al.* Acid-assisted ligand exchange enhances coupling in colloidal quantum dot solids. *Nano Letters.* **18**, 4417-4423 (2018).
16. Aqoma H, Jang S-Y. Solid-state-ligand-exchange free quantum dot ink-based solar cells with an efficiency of 10.9%. *Energy Environ. Sci.* **11**, 1603-1609 (2018).
17. Kirmani AR, *et al.* Overcoming the ambient manufacturability‐scalability‐performance bottleneck in colloidal quantum dot photovoltaics. *Adv. Mater.* **30**, 1801661 (2018).
18. Song JH, Choi H, Kim Y-H, Jeong S. High performance colloidal quantum dot photovoltaics by controlling protic solvents in ligand exchange. *Adv. Energy Mater.* **7**, 1700301 (2017).
19. Pradhan S, Stavrinadis A, Gupta S, Bi Y, Di Stasio F, Konstantatos G. Trap-state suppression and improved charge transport in PbS quantum dot solar cells with synergistic mixed-ligand treatments. *Small.* **13**, 1700598 (2017).
20. Azmi R, *et al.* Highly efficient air-stable colloidal quantum dot solar cells by improved surface trap passivation. *Nano Energy.* **39**, 86-94 (2017).

21. Aqoma H, *et al.* High-efficiency photovoltaic devices using trap-controlled quantum-dot ink prepared via phase-transfer exchange. *Adv. Mater.* **29**, 1605756 (2017).
22. Zhao M, *et al.* High hole mobility in long-range ordered 2D lead sulfide nanocrystal monolayer films. *Adv. Funct. Mater.* **26**, 5182-5188 (2016).
23. Cao Y, Stavrinadis A, Lasanta T, So D, Konstantatos G. The role of surface passivation for efficient and photostable PbS quantum dot solar cells. *Nat. Energy.* **1**, 1-6 (2016).
24. Malgras V, Nattestad A, Yamauchi Y, Dou SX, Kim JH. The effect of surface passivation on the structure of sulphur-rich PbS colloidal quantum dots for photovoltaic application. *Nanoscale.* **7**, 5706-5711 (2015).
25. Zherebetskyy D, *et al.* Hydroxylation of the surface of PbS nanocrystals passivated with oleic acid. *Science.* **344**, 1380-1384 (2014).
26. Yang ZY, *et al.* Mixed-quantum-dot solar cells. *Nat. Commun.* **8**, 1-9 (2017).
27. Proppe AH, *et al.* Picosecond charge transfer and long carrier diffusion lengths in colloidal quantum dot solids. *Nano Lett.* **18**, 7052-7059 (2018).
28. Nakazawa N, *et al.* The interparticle distance limit for multiple exciton dissociation in PbS quantum dot solid films. *Nanoscale Horiz.* **4**, 445-451 (2019).
29. Liu F, *et al.* Near−infrared emission from tin–lead (Sn–Pb) alloyed perovskite quantum dots by sodium doping. *Angew. Chem.*, **132**, 8499-8502 (2020).
30. Zhang Y, Wu G, Liu F, Ding C, Zou Z, Shen Q. Photoexcited carrier dynamics in colloidal quantum dot solar cells: Insights into individual quantum dots, quantum dot solid films and devices. *Chem. Soc. Rev.* **49**, 49-84 (2020).
31. Liu Y, Kim D, Morris OP, Zhitomirsky D, Grossman JC. Origins of the stokes shift in PbS quantum dots: Impact of polydispersity, ligands, and defects. *ACS Nano.* **12**, 2838-2845 (2018).
32. Voznyy O, *et al.* Origins of stokes shift in PbS nanocrystals. *Nano Lett.* **17**, 7191-7195 (2017).
33. Anderson NC, Hendricks MP, Choi JJ, Owen JS. Ligand exchange and the stoichiometry of metal chalcogenide nanocrystals: Spectroscopic observation of facile metal-carboxylate displacement and binding. *J. Am. Chem. Soc.* **135**, 18536-18548 (2013).
34. Zhang L, Wu L, Wang K, Zou B. Pressure-induced broadband emission of 2D organic-inorganic hybrid perovskite $(C_6H_5C_2H_4NH_3)_2PbBr_4$. *Adv. Sci.* **6**, 1801628 (2019).
35. Jones M, Lo SS, Scholes GD. Quantitative modeling of the role of surface traps in CdSe/CdS/ZnS nanocrystal photoluminescence decay dynamics. *Proc. Natl. Acad. Sci.*. **106**, 3011-3016 (2009).
36. Sizov F, Plyatsko S. Homogeneity range and nonstoichiometric defects in IV–VI narrow-gap semiconductors. *J. Cryst. Growth.* **92**, 571-580 (1988).
37. Gilmore RH, *et al.* Epitaxial dimers and auger-assisted detrapping in PbS quantum dot solids. *Matter.* **1**, 250-265 (2019).
38. Hughes BK, *et al.* Synthesis and spectroscopy of PbSe fused quantum-dot dimers. *J. Am. Chem. Soc.* **136**, 4670-4679 (2014).
