# Supplementary material for: The effect of water on colloidal quantum dot solar cells
Source: Nat Commun. 2021 Jul 19;12:4381. doi: 10.1038/s41467-021-24614-7 (PMC8289876; doi:10.1038/s41467-021-24614-7)
Supplement: Supplementary file 2 — Solar Cells Reporting Summary [file 41467_2021_24614_MOESM2_ESM.pdf]

nature research

Corresponding author(s): Zeke Liu, Wanli Ma.

# Solar Cells Reporting Summary

Nature Research wishes to improve the reproducibility of the work that we publish. This form is intended for publication with all accepted papers reporting the characterization of photovoltaic devices and provides structure for consistency and transparency in reporting. Some list items might not apply to an individual manuscript, but all fields must be completed for clarity.

For further information on Nature Research policies, including our data availability policy, see Authors & Referees.

## Experimental design

**Please check: are the following details reported in the manuscript?**

### 1. Dimensions

| Item | Yes | No | Details |
|---|---|---|---|
| Area of the tested solar cells | ☒ | ☐ | 0.0725 cm^2, described in "Methods" section. |
| Method used to determine the device area | ☒ | ☐ | The overlap of ITO and gold electrodes. The area was verified by microscope. |

### 2. Current-voltage characterization

| Item | Yes | No | Details |
|---|---|---|---|
| Current density-voltage (J-V) plots in both forward and backward direction | ☐ | ☒ | No hysteresis was observed for all CQD devices. The hysteresis-free behavior in CQD PV is also widely-accepted in literatures. |
| Voltage scan conditions<br>*For instance: scan direction, speed, dwell times* | ☒ | ☐ | Described in "Methods" section. Scan direction:-0.8 V to 0.8 V; Speed: 0.01 V per point; Swell times: 10 ms. |
| Test environment<br>*For instance: characterization temperature, in air or in glove box* | ☒ | ☐ | The devices were measured under ambient air at room temperature. |
| Protocol for preconditioning of the device before its characterization | ☐ | ☒ | No need for encapsulation unless it is specifically mentioned. |
| Stability of the J-V characteristic<br>*Verified with time evolution of the maximum power point or with the photocurrent at maximum power point; see ref. 7 for details.* | ☐ | ☒ | Not applicable. |

### 3. Hysteresis or any other unusual behaviour

| Item | Yes | No | Details |
|---|---|---|---|
| Description of the unusual behaviour observed during the characterization | ☐ | ☒ | No hysteresis was observed for all CQD devices. The hysteresis-free behavior in CQD PV is also widely-accepted in literatures. |
| Related experimental data | ☐ | ☒ | No hysteresis or any other unusual behavior. Please see above. |

### 4. Efficiency

| Item | Yes | No | Details |
|---|---|---|---|
| External quantum efficiency (EQE) or incident photons to current efficiency (IPCE) | ☒ | ☐ | Supplementary Figure 22. |
| A comparison between the integrated response under the standard reference spectrum and the response measure under the simulator | ☒ | ☐ | Table 2. |
| For tandem solar cells, the bias illumination and bias voltage used for each subcell | ☐ | ☒ | N.A. |

### 5. Calibration

| Item | Yes | No | Details |
|---|---|---|---|
| Light source and reference cell or sensor used for the characterization | ☒ | ☐ | It is described in “Methods” section of the main text. |
| Confirmation that the reference cell was calibrated and certified | ☒ | ☐ | It is described in “Methods” section of the main text. |



November 2017

Calculation of spectral mismatch between the reference cell and the devices under test

☐ Yes
☒ No

No mismatch calculation was performed.

6. Mask/aperture

Size of the mask/aperture used during testing

☐ Yes
☒ No

Mask/aperture is not used.

Variation of the measured short-circuit current density with the mask/aperture area

☐ Yes
☒ No

Mask/aperture is not used.

7. Performance certification

Identity of the independent certification laboratory that confirmed the photovoltaic performance

☐ Yes
☒ No

A certified efficiency is not relevant for the scope of this work.

A copy of any certificate(s)
*Provide in Supplementary Information*

☐ Yes
☒ No

No certificate. Please see above.

8. Statistics

Number of solar cells tested

☒ Yes
☐ No

Table 2

Statistical analysis of the device performance

☒ Yes
☐ No

Table 2.

9. Long-term stability analysis

Type of analysis, bias conditions and environmental conditions
*For instance: illumination type, temperature, atmosphere humidity, encapsulation method, preconditioning temperature*

☒ Yes
☐ No

The stabilities obtained under humid and dry air at room temperature or 85 were shown in Figure 4f.
